# Patient and surgeon perspectives of a large-scale system for automated, real-time monitoring and feedback of shared decision-making integrated into surgical practice: a qualitative study

**DOI:** 10.1136/bmjopen-2025-099090

**Published:** 2025-06-27

**Authors:** Christin Hoffmann, Kerry N L Avery, Rhiannon C Macefield, Val Snelgrove, Leila Rooshenas, Hilary L Bekker, Della Hopkins, Christie Cabral, Jane M Blazeby, Ben Gibbison, Shireen Hickey, Adam Williams, Jon Aning, Andrew Judge, Andrew Smith, Archana Lingampalli, Barnaby Reeves, Jessica Preshaw, Michael R Whitehouse, Paul Cresswell, Philip Braude, Shelley Potter, Timothy Beckitt, Timothy Whittlestone, Angus G K McNair

**Affiliations:** 1 NIHR Bristol Biomedical Research Centre, University Hospitals Bristol and Weston NHS Foundation Trust and University of Bristol, Bristol, UK; 2 Patient Representative, Bristol, UK; 3 Leeds Unit of Complex Intervention Development (LUCID), Leeds Institute of Health Sciences, School of Medicine, University of Leeds, Leeds, UK; 4 The Research Centre for Patient Involvement (ResCenPI), Department of Public Health, Aarhus Universitet, Aarhus, Central Denmark Region, Denmark; 5 North Bristol NHS Trust, Bristol, UK; 6 Centre for Academic Primary Care, Bristol University, Bristol Medical School, Bristol, UK; 7 University Hospitals Bristol and Weston NHS Foundation Trust, Bristol, UK; 8 Bradford Institute for Health Research, Bradford Teaching Hospitals NHS Foundation Trust, Bradford, UK; 9 Bristol Urological Institute, Southmead Hospital, North Bristol NHS Trust, Bristol, UK; 10 Population Health Sciences, Bristol Medical School, University of Bristol, Bristol, UK; 11 Translational Health Sciences, Bristol Medical School, University of Bristol, Bristol, UK; 12 Bristol Trials Centre, Population Health Sciences, University of Bristol, Bristol Medical School, Bristol, UK

**Keywords:** Decision Making, SURGERY, Health Services, Health policy, QUALITATIVE RESEARCH

## Abstract

**Objective:**

To explore patient and healthcare professional perceptions about the acceptability and impact of a large-scale system for automated, real-time monitoring and feedback of shared decision-making (SDM) that has been integrated into surgical care pathways.

**Design:**

Qualitative, semistructured interviews were conducted with patients and healthcare professionals between June and November 2021. Data were analysed using deductive and inductive approaches.

**Setting:**

Large-scale monitoring of SDM has been integrated in NHS surgical care across two large UK National Health Service Trusts.

**Participants:**

Adult surgical patients (N=18, 56% female), following use of an SDM real-time monitoring and feedback system, and healthcare professionals (N=14, 36% female) involved in their surgical care. Patient recruitment was conducted through hospital research nurses and professionals by direct approach from the study team to sample individuals purposively from seven surgical specialties (general, vascular, urology, orthopaedics, breast, gynaecology and urgent cardiac).

**Results:**

10 themes were identified within three areas of exploration that described factors underpinning: (1) the acceptability of large-scale automated, real-time monitoring of SDM experiences, (2) the acceptability of real-time feedback and addressing SDM deficiencies and (3) the impact of real-time monitoring and feedback. There was general support for real-time monitoring and feedback because of its perceived ability to efficiently address deficiencies in surgical patients’ SDM experience at scale, and its perceived benefits to patients, surgeons and the wider organisation. Factors potentially influencing acceptability of large-scale automated, real-time monitoring and feedback were identified for both stakeholder groups, for example, influence of survey timing on patient-reported SDM scores, disease-specific risks, patients’ dissatisfaction with hospital processes. Factors particularly important for patients included concerns over digital exclusion exacerbated by electronic real-time monitoring. Factors unique to professionals included the need for detailed, qualitative feedback of SDM to contextualise patient-reported SDM scores.

**Conclusions:**

This study explored factors influencing the acceptability of automated, real-time monitoring and feedback of patients’ experiences of SDM integrated into surgical practice, at scale among key stakeholders. Findings will be used to guide refinement and implementation of SDM monitoring and feedback prior to formal development, evaluation and implementation of an SDM intervention in the NHS.

**Trial registration number:**

ISRCTN17951423.

**The original protocol:**

doi: 10.1136/bmjopen-2023-079155.

**STRENGTHS AND LIMITATIONS OF THIS STUDY**

- A strength of this study is its methodologically robust approach to explore the perspectives of both patients and healthcare professionals.
- Factors underpinning acceptability and impact of a system for automated, real-time monitoring and feedback of shared decision-making (SDM) were identified to inform its large-scale implementation and intervention development to improve SDM at scale.
- Limitations with regard to diversity and inclusivity have been identified that are being addressed in ongoing work, which examines the views of underserved groups on real-time SDM monitoring and feedback.

## Introduction

 Shared decision-making (SDM) is a key pillar of patient-centred care^1 2^ and can impact health outcomes.^3^,^6^ High-quality SDM discussions for many surgical treatment decisions are particularly important, especially those that are preference-sensitive, because consequences are usually irreversible.^7^ About one-third of surgical patients, however, report deficiencies with SDM experiences.^8^ Improving surgical care pathways to enable consistent SDM practices. SDM remains a key patient and policy priority in many modern healthcare systems.^1 9 10^

Health policy recommends a comprehensive approach to improving SDM in practice. It suggests a combination of interventions to support patients, professionals and organisations, for example, use of patient decision aids, provision of healthcare professional (HCP) training and supportive leadership.^11 12^ Routine measurement and evaluation of patient-reported SDM within healthcare organisation systems has been identified as essential for driving these improvements, with financial incentives promoting its uptake.^13 14^ Although previous UK service improvement programmes incorporated SDM measurement in specific healthcare contexts,^15^,^17^ limited evidence exists for how to effectively integrate, monitor and sustain SDM measurement at scale.^3 12^ In addition, feedback of patient-reported measures, either as a key intervention component or on its own, is a widely used quality improvement intervention^18 19^ and has been shown to positively impact patient care and outcomes in other contexts.^20^,^22^ For example, a trial evaluating integrated, real-time symptom monitoring with feedback to clinical teams has led to improvements in overall 2-year survival in advanced cancer patients by enabling clinicians to respond timely to patient-reported symptoms and preventing downstream adverse complications.^23^ Similar interventions in chronic disease patients improved outcomes due to early detection of problems.^24^ Therefore, it is plausible that measuring patient experiences of SDM can detect deficiencies early, and feedback to clinicians can be promising mechanisms to prompt efficient remediation of problems, improving SDM experiences before surgery. The optimal approach for feedback design and implementation, however, remains to be explored. A better understanding of key stakeholders’ perspectives can optimise the design of feedback mechanisms to drive meaningful and sustained behaviour change within clinical teams along the surgical care pathway.^25^,^27^

We integrated within surgical care pathways of two National Health Service (NHS) trusts, a novel electronic system to monitor and feedback surgical patients’ experience of SDM automatically and in real-time.^28 29^ The system has the potential to support large-scale interventions to enhance patients’ experiences of SDM before surgery and to ultimately improve patient and health service outcomes.^4 6^ In-depth exploration, using qualitative methods, of stakeholders’ views towards new systems is important to inform organisation-wide implementation.^30^,^33^ Surgical patients and surgeons are key stakeholders and primary intended users of real-time SDM monitoring and feedback. Improved understanding of their perceptions of the system and factors influencing its acceptability is necessary to facilitate its effective implementation and intervention development to improve SDM.

### Aim

To explore patient and HCP perceptions about the acceptability and impact of a system for automated, real-time monitoring and feedback of SDM integrated into elective surgical care pathways in the NHS.

## Methods

This qualitative study was guided by the interpretivist paradigm which epistemologically and ontologically acknowledges multiple realities.^34^ It employed semistructured interviews to elicit views and experiences of patient and HCP participants and applied codebook thematic analysis to generate themes.^35^ Conduct and reporting of this study followed the Consolidated criteria for Reporting Qualitative research checklist.^36^

### Study context

This study was part of a larger programme of work (the ALPACA study) that seeks to codevelop a decision support intervention to improve SDM practices within surgical pathways.^28^ The intervention is expected to use a system for automated and real-time monitoring and feedback of patients’ experiences of SDM before surgery. It is designed for broad integration across multiple surgical specialties to support organisation-level improvements in SDM by enabling scalable, routine SDM measurement across a large range of surgical decision contexts, including, for example, cancer or benign, and more or less preference-sensitive treatment decisions. Details about the system and processes for its integration into surgical practice are available elsewhere^28 29^ and briefly described below.

Incorporating routine data into clinical practice is a multistep process involving systematic measurement and data collection, analysis, interpretation and integration into clinical care. Our protocol describes this process as ‘SDM monitoring and feedback’. Integrating real-time SDM monitoring in surgical care pathways was achieved through procuring and customising an off-the-shelf electronic patient-reported outcome software (Cemplicity, New Zealand) in two NHS Trusts in England (North Bristol Trust (NBT), University Hospitals Bristol and Weston Foundation Trust (UHBW)) from April 2021. In collaboration with the software provider, Structured Language Queries were developed to enable secure, automated, daily data transfers between the system and hospital data warehouse to monitor and feedback surgical patients’ experiences. Monitoring involved the system automatically administering validated, electronic patient-reported SDM instruments via text message or email to patients within 24 hours of elective surgery booking, following SDM discussions. Patients’ responses were received in real-time and securely returned to NHS Trust Electronic Patient Records. Feedback involved the system processing, analysing and displaying patients’ responses in an electronic dashboard accessible to the clinical teams.

The participating hospitals are large tertiary care centres in the South West of England, UK. NBT is among the largest acute NHS trusts in the UK, offering a comprehensive range of acute clinical care to both local and regional clinical commissioning groups in South West of England. Surgical departments where the system for real-time SDM monitoring and feedback has been integrated included services in general surgery, urology, gynaecology, orthopaedic, breast and vascular surgery. One of UHBW’s departments was included as the regional cardiac surgical centre for the South West of England.

Data were collected for NHS quality improvement purposes with approval from Trust clinical governance committees.

### Participants, sampling and recruitment

#### Patient participants

Patient participants were recruited from the two NHS Trusts (NBT, UHBW) where the system for real-time monitoring and feedback of SDM had already been integrated into surgical practice.

Patients were eligible if they used the electronic platform to complete an SDM measure as part of the wider study. The electronic platform invited all adult patients booked for elective surgery across general, gynaecological, breast, urological, orthopaedic or vascular surgical departments (NBT) and urgent cardiac surgery (UHBW). Patients were excluded if they lacked decisional capacity for medical treatments, had undergone unplanned (emergency) surgery or were booked for outpatient procedures (eg, gastrointestinal and urological endoscopy) because these decision contexts differ from those typically required to achieve high-quality SDM, including the ability and time for deliberation, the availability of multiple options and the involvement of both the patient and professional in making the treatment choice.^7^

A purposive sampling strategy was adopted to ensure that insights are drawn from a range of perspectives. Sampling characteristics considered a priori included variation by (1) experiences of different types of surgery (eg, general, vascular, urology, orthopaedics, breast, gynaecology, urgent cardiac), (2) experience of SDM process (ie, good/poor experience as determined by SDM scores) and (3) sociodemographic characteristics (ie, sex, age, ethnicity). Available sociodemographic characteristics of participants were reviewed as recruitment progressed to adjust recruitment efforts to purposively sample individuals with different experiences, offering potentially varied perspectives. No further sociodemographic details were collected to retain the anonymity of participants. Data on the number of participants declining to take part in interviews were not collected.

Potential patient participants were identified and contacted by a member of hospital staff (eg, research nurse) with authorised access to patient data and the system for real-time SDM monitoring and feedback. Contact was initiated via email or telephone to explain the study, send the patient information sheet and consent form and to ask for permission to pass on their contact details to a researcher. If patients agreed and expressed interest in participating, a member of the study team (CH) followed up with a phone call to answer any queries and arrange a suitable interview date.

Recruitment stopped when no new codes or meanings were identified through analysis of additional transcripts. This decision was supported by regular multidisciplinary team discussions which involved reviewing whether the identified codes/themes provided sufficient conceptual depth to answer the research question (see analysis section for more details).^37^,^39^

#### Professional participants

HCPs were consultant surgeons working in the participating surgical departments. Included were those that: (1) booked eligible patients for surgery, (2) were involved in SDM discussions with eligible patient participants or (3) had overall responsibility for eligible patients’ care.

Eligible HCPs were identified through their involvement in surgical teams within the two NHS Trusts and approached by the principal investigator (AGKM). Contact was initiated through face-to-face conversations in the hospital or clinical departmental meetings where the project was presented, inviting HCPs to take part in interviews. Follow-up emails including a participant information sheet and consent form were sent to HCPs and teams expressing interest in arranging a suitable time and date for an interview.

A purposive sampling approach sought variation by surgical specialty and sex. No information about other personal details (eg, ethnicity, age) or those declining the interview was recorded. Recruitment continued until sufficient conceptual depth was achieved as outlined above.

### Data collection

Semistructured, one-to-one interviews with participants were undertaken remotely using video conferencing software (Zoom, MS Teams) or telephone. All interviews were audio-recorded and conducted in English by one of two authors (AGKM, a methodologist and academic surgeon not involved in the care of participants; CH, a social scientist with extensive experience of health services research). Both interviewers are trained in qualitative methods and have extensive experience conducting qualitative research in secondary care settings and for health services research.

Patients received a participant information sheet and completed an electronic consent form prior to the interview. Participants were made aware of the study purpose, aims and why they were invited to participate. Interviewers introduced themselves to explain their occupation and role in the study. It was emphasised that in case the patient participant raised any issues about their clinical care, the interviewees were unable to address these, but could direct them back to the clinical team.

Topic guides, developed separately for patient and professional stakeholders, were piloted and refined before interviews commenced (online supplemental file 1). Topic guides focused on two areas of exploration aligned with the study aim: acceptability of real-time monitoring and feedback and impact of this on clinical care. These were defined a priori as topics of interest to support the wider ALPACA study.^28^

### Analysis

Anonymised interview transcripts were uploaded to a qualitative data management software (NVivo, V.14). The analytical process followed principles of deductive and inductive coding^40^ and template analysis^41^ and was performed in parallel to data collection to prospectively inform ongoing participant sampling. Data analyses were performed separately and sequentially for each stakeholder group (HCPs, patients) to allow exploration of any potential differences within their experiences and context. Analyses were performed by two trained and experienced qualitative researchers (AGKM and CH).

The analytical process involved the two researchers separately (1) reading the transcripts several times; (2) deductively coding participants’ responses according to the two broad areas of exploration (ie, the acceptability of real-time SDM monitoring and feedback and impact on clinical practice); (3) generating initial codes within the two broad areas of exploration, by assigning latent and semantic labels to relevant excerpts and drawing on constant comparison^42^; (4) generating early themes by collating similar codes and defining an initial coding template^41^ and (5) iteratively refining the coding template and early themes through interpretation of the data and by triangulating views through multidisciplinary study team discussions. Themes were further developed through analysis of more sets of transcripts, applying and further modifying the coding template as necessary.

Summaries (descriptive reports) of findings were written for each stakeholder group following rounds of transcript analyses and discussed within the multidisciplinary study team and patient advisory group. Discussions were used to enrich interpretations of the data by exploring new lines of enquiry considered important to addressing the study objectives. The summaries were iteratively developed as analyses proceeded. The separate reports were then synthesised to form the basis for research outputs by comparing and contrasting participants’ accounts within and between stakeholder groups. Thereby, themes with similar conceptual meaning were combined and theme labels harmonised; themes that were identified as conceptually distinct and unique to either patient or professional participants were kept separate.

### Patient and public involvement

A dedicated patient and public advisory group was established for the wider ALPACA study.^28^ Responsibilities included oversight of study and patient and public involvement and engagement activities to ensure these are aligned with patient priorities. The group was involved in interpreting the results following initial analyses. The patient coauthor (VS) codeveloped topic guides and recruitment materials for this study and was involved in writing this manuscript.

## Results

### Participant characteristics

A total of 18 patient and 14 professional participants were interviewed. Interviews were conducted between June and November 2021 and lasted between 25 and 57 min. One patient participant consented but did not attend the interview. All professional participants who consented to take part proceeded with the interview.

Participant characteristics are summarised in table 1. Patient participants were 56% female, and all from a white British background. They were on average 52 years old, and their surgical experiences spanned a total of seven surgical specialties. HCPs were 64% male and represented consultant surgeons from three surgical specialties.

**Table 1:** Participant characteristics

Characteristic	Patient participants (N=18)	Professional participants (N=14)
Age (years), mean (±SD)	52 (±14)	Not reported
Sex, n		
Female	10	5
Male	8	9
Ethnicity, n		
White British	18	Not reported
Specialty, n		
Urology	5	7
General	4	3
Breast	3	–
Orthopaedics	2	–
Urgent cardiac	2	–
Vascular	1	4
Gynaecology	1	–

### Themes identified from interviews

A total of 10 themes relating to participants’ perceptions were identified across three areas of exploration (acceptability of real-time monitoring, acceptability of real-time feedback and addressing SDM deficiencies, impact on clinical practice). An overview of themes and summaries of findings per theme are displayed in table 2. Detailed descriptions of each theme, including illustrative quotes, are provided below. Anonymised quotes were labelled with a unique participant ID, using acronyms ‘PAT’ (patient participant) and ‘HCP’ (healthcare professional) to distinguish their participant groups.

**Table 2:** Overview of areas of exploration, identified themes and summary of findings

Area of exploration		Themes	Summary of findings
Acceptability	Real-time monitoring of SDM experiences	Benefits and challenges in identifying SDM deficiencies	All participants expressed support for a real-time monitoring system to assess SDM experiences and its ability to detect deficiencies in SDM experiences. Professionals discussed engagement-related challenges.
		Complexities of SDM measurement	Both participant groups discussed the perceived complexities of reliably measuring SDM and pointed out common sources of variability of SDM experiences that might influence patient-reported SDM scores.
		Challenges of distributed decision making	Some participants in both participant groups discussed difficulties in tracking and attributing SDM experiences in complex care pathways involving multiple healthcare providers.
		Limited inclusivity of real-time monitoring	Both participant groups expressed concerns regarding digital and language inclusivity, with patients frequently highlighting limitations for elderly, ethnic minority and disabled patients.
	Real-time feedback and addressing SDM deficiencies	Balanced and informative feedback	Both participant groups highlighted the importance of sharing positive and negative feedback, with professionals emphasising the need for details about reasons for poor SDM experiences.
		Approaches to providing feedback to clinical teams	Professionals expressed their preference for a ‘triage process’ to providing feedback to clinical teams, whereby data about patient-reported SDM scores are initially processed and filtered by intermediaries before involving surgeons, only where necessary.
		Approaches to responding to feedback	Both participant groups considered a follow-up encounter to be an appropriate response when patient-reported SDM scores suggest deficiencies in SDM experiences, emphasising that tailored discussions need to address individual concerns.
Impact on clinical practice		Perceived impact on patients	All participants stressed the important role of the system in promoting patient-centred care, with patients discussing specific benefits of decision satisfaction and reassurance.
		Perceived impact on surgeons	Participants perceived the system as beneficial for identifying practice improvements and training needs, with professionals expressing concern about workload and emotional burden.
		Perceived impact on department or organisation	Professionals discussed the potential of the system for identifying and addressing systemic performance issues, highlighting benefits such as trend analysis and organisational reputation.

SDM, shared decision-making.

### Acceptability of real-time monitoring of SDM experiences

#### Benefits and challenges in identifying SDM deficiencies

Participants expressed overall support for electronic, automated real-time monitoring of patients’ SDM experiences. Participants highlighted the benefits of the electronic survey, such as “*it was quite quick*” (PAT004), *“very easy to complete”* (PAT015) and “*seems efficient*” (HCP004), and “*straightforward and simple to use*”(HCP013), which support the acceptability of SDM monitoring.

It was widely recognised that monitoring has the potential to detect SDM deficiencies and serves as a crucial first step towards improving SDM experiences.

I want that opportunity to be able to discuss exactly what’s involved and you know if that can happen as a result of this survey then that’s great. (PAT003)

I received the survey without knowing they had made a decision to have an operation. […] (PAT018)

It seemed one of those projects that you think how do we not know that? How have we not asked this before? Why are we just steaming ahead with information leaflets and bespoke consent forms with all the risks written out and yet we didn’t ask them whether it was what they wanted rather than what a medico legal team thought we should do. So it’s a critical project. (HCP006)

Patient participants often cited an interest in giving feedback and thereby potentially improving care for future patients as important drivers for completing SDM measurement. Interviewees often noted that reporting SDM experiences can lead to feeling valued and listened to.

I think most people would fill out a survey like this, especially for, if it’s on their own health. (PAT004)

Unless you were part of doing a survey on something like this, you never really get to tell people your experience, whether it be a good one, whether it be a bad one, you might get somebody who has had the most horrendous time (PAT007)

Again, being taken seriously, so if there is a real, serious problem, sometimes it’s not heard, it’s not listened to and I feel […] that it’s going to help in the future (PAT002)

Some professional participants also commented on the wide applicability of real-time SDM monitoring beyond surgery, demonstrating the perceived potential to enhance SDM in various clinical settings.

It could be extended to the non-operative cases (HCP008).

So what’s good about it is it’s rapid, the questionnaire is short, […] it’s not complex and the questions are quite well designed. If you look at them you think you know what, actually they have wide applicability. (HCP001)

Professional participants emphasised the importance of HCP buy-in for the successful uptake and acceptance of a system for real-time SDM monitoring. They highlighted the need for engagement strategies that address concerns such as time pressure and fear of SDM monitoring being perceived as a tool for assigning blame for deficiencies in decision-making.

[D]o it in a way that doesn’t make time pressured, angry surgeons more angry (HCP003)

What we’ve got to do is engage the clinicians in the fact that it’s not a blame culture (HCP001)

The importance of engaging all clinical team members during the implementation process was also apparent in one patient’s experience who reported: *“The ward doctor was surprised I got the survey because he didn’t know you did surveys”* (HCP018).

#### Complexities of SDM measurement

Both participant groups noted complexities of real-time SDM monitoring and discussed factors that can impact perceived reliability of the data. Participants shared their concern about SDM being influenced by the context in which care is provided and varying preferences for decision styles. Most commonly, procedure/disease and specialty-dependency were highlighted. In particular, participants stressed the importance of SDM for elective surgery and operations that carry the greatest risks for and impacts on patients’ quality of life.

All of us [patients] don’t necessarily want to question the decisions of those who have positions which are not equal to our own do we. […] I think it will depend on how patients feel about their consultations and where in the cycle of treatment they get it because there’s no doubt at all if you’re looking at cancer it’s very emotive. Surgery often takes place very quickly […] so it’s how you catch people and that will be quite individual in some ways. (PAT001)

[…]bigger operations have higher risk, it’s more important that those patients are engaged in the process, particularly for what we do. Carotid endarterectomy [surgery to unblock a main blood vessel], an operation that prevents something that might or might not happen, shared decision-making is crucial. (HCP001)

About half of patient participants also mentioned other sources of potential variability in patient-reported SDM experiences which may influence responses to real-time data capture. Participants discussed the importance of the timing of the SDM survey, suggesting that patients might feel too unwell or distressed at various points in time before surgery.

I think probably if its straight after the consultation or before the operation, I mean a lot of these people are… well I know I was, I was really ill and probably giving your time to do that is probably not a high priority. (PAT005)

Patients not wanting to criticise their care team or potentially affecting care (see also theme 2.1) was mentioned as another factor that may influence responses.

My mum is a very quiet person and she wouldn’t push forward for anything, you know, she wouldn’t go, well actually I’m not happy about that, and there are lots of people like that and they may not want it to rock the boat or cause problems when they have surgery. (PAT002)

All professional participants raised concerns that poor patient-reported experiences might not always reflect actual SDM deficiencies, potentially affecting surgeons’ acceptability of SDM monitoring. They noted that misinterpretation of survey questions or unrelated frustrations (eg, with hospital processes) could influence patients’ survey responses.

I think patients can just lump everything […] into one big umbrella and […] saying that they’re not happy about shared decision-making, actually if you unpick it, it might be more about the practicalities of the booking process or the kind of logistics (HCP010)

It’s not always that it is a poor experience as such, it’s just that you might have worked on a slightly different agenda to them or you may not have said what they wanted you to say. (HCP003)

[I]f a patient’s told that they have to have an operation, then they have to think about the consequences (but) they might not instantly think about all that and have all their questions ready and then they might become unhappy that they’ve forgot to or it was sprung on them. (HCP008)

Professional participants also highlighted broader challenges in capturing patients’ SDM experiences by acknowledging *“It can be a difficult thing to measure”* (HCP007). Interviewees drew comparisons to other patient-reported outcome measures and associated potential difficulties with interpreting scores.

I still think it is difficult because if you give three patients a pain score who have the same pain, they will score differently on the pain scales because some of them will be more bothered by it and others will be less bothered by it. (HCP012)

#### Challenges of distributed decision making

Though discussed by fewer participants, both participant groups talked about challenges caused by distributed decision-making contexts that could affect stakeholder acceptability of SDM monitoring. This means care pathways where patients are under the care of multiple HCPs and providers (eg, general practitioner, oncologist, nurse, surgeon) and where the SDM experience cannot be isolated to a single event. This leads to challenges in identifying where SDM problems have occurred and what/who they can be attributed to. For example, patients may be referred from other hospitals or are on pooled operating lists where multiple healthcare encounters influence the decision-making experience.

The whole feeling with this whole gynae thing, is that it doesn’t feel very centralised, you're dealing with the physio, you go and see a nurse for the pessary, you just don’t feel like it’s very joined together. (PAT017)

My worry about urology for example is about our pooled list system […] I don’t really want to be getting alerts when one of my other colleagues has not had a good interaction with a patient in the one stop clinic. (HCP010)

A perceived lack of communication between institutions and teams ultimately led to a feeling of uncertainty about whether their health problems were understood and doubt whether surgery was really the best option. A small number of patients explained how discrepancies or delays in information provision caused by different teams or institutions may impact their SDM experience, which may affect patient-reported experiences.

To be honest, up until the phone call—up until six weeks before I was due for my op, I've had a little contact with the doctor, I've had very little information. I’ve been given probably three different diagnoses of what was wrong with my knee. (PAT015)

#### Limited inclusivity of real-time monitoring

Both participant groups commonly discussed barriers to digital and language inclusivity common to accessing electronic, real-time monitoring systems in a healthcare setting.

Patient participants frequently highlighted limitations in relation to patients’ age, preferred language and disability status, which can increase difficulty engaging in real-time SDM monitoring without the synchronous provision of additional support.

I think the challenges would be people’s ability to use technology. Moving forward there are people of any age group who still can’t, especially if you have got people with special needs. […] There are obviously sectors in society where they need more care in a situation like that, because it is very frightening, so filling out questionnaires beforehand, they are always gonna need help. […] when you have got elderly or people with a disability or a learning disability, their interpretation of things can cause a stressful situation (PAT007)

Professional participants also raised concerns about potentially encouraging health-related inequalities for a range of under-served groups.

People with learning disabilities, people with mental health issues, people whose literacy levels are not high might struggle with this. (HCP004)

One of the concerns that I have with any form of patient reported measures […] they lead to an increase in health inequality and I think there is a little bit of evidence for that as well is that those who shout loudest, tend to get the most input and those who are quietest who are often those that need the most input (HCP011)

Some suggested the lack of inclusivity needs addressing in later stages of implementing real-time monitoring of SDM to avoid exacerbating inequalities in future healthcare provision.

I think the only downside for me is the health and equalities possibly, that you’re not reaching the hard to reach groups, so that’s the only downside but it’s still better than doing nothing which is what we do now so, even if you don’t reach everyone, it’s still better than nothing. (HCP004)

The problem of course is that we’re using an electronic format and we are therefore serving ourselves up to patients who’ve got Smartphones, who are electronically engaged, who speak good English and therefore largely the middle-classes. […] So we by definition have a huge sampling error in this and […] it’s going to have to be a project priority at some point to identify those patients and think about another intervention (HCP001)

### Acceptability of real-time feedback and addressing SDM deficiencies

#### Balanced and informative feedback

Both participant groups considered it appropriate to provide feedback on patients’ personal SDM experience and stressed the importance that SDM deficiencies should be known.

Yes, I would like them to know how I feel about it and my concern. (PAT008)

I’m terribly concerned about the individual. So, if a person hadn’t had a good experience, I would want to put it right for that individual. (HCP009)

I think from a personal perspective, if you’d seen that patient, you’d want to know which areas they felt the decision making was deficient in. (HCP002)

The need to share positive alongside negative feedback was highlighted by stakeholders in different ways. Patient participants raised concerns that providing negative feedback may cause distress and additional workload for HCPs or could negatively affect their own care.

I don’t want to put pressure on anybody that’s under pressure as it is. […] It’s a worry because you do feel, so if I say this, will they not do my surgery? Will they take it to heart and you know, or will I cause stress and anguish with the surgical team? (PAT002)

Professional participants also suggested that balancing positive and negative messages can impact acceptability in real-time monitoring and feedback among surgeons who demonstrate lower engagement.

I think the other thing is not to forget positive feedback can be as important as negative. So actually, as a clinician, occasionally getting a positive feedback is also very nice. I think, particularly as surgeons, we are very good at picking up when someone else has done something wrong. (HCP005)

The problem is that you get a lot of egotistical characters, and I think that a lot of times the base response will be ‘I didn’t do anything wrong.’ So, you’ve got to make sure that information is coming across in a way that you're not reinforcing that that initial reaction. (HCP007)

Professional participants unanimously agreed that receiving patient-reported scores from real-time SDM monitoring is helpful to contextualise responses, but numerical data alone are insufficient. They emphasised the need for qualitative detail about the reasons for patients’ low scores to understand and address any deficiencies in patients’ SDM experience. Some interviewees also suggested a call to action in feedback to facilitate further action.

[These] scores mean nothing to the clinician. (HCP001)

I think it needs to be more descriptive. Yes, you can give the overall number as well but I still think you need more description one way or another. (HCP012)

I suppose some detail as to what made that an unsatisfactory experience for the patient and also what to do next. (HCP008)

#### Approaches to providing feedback to clinical teams

Professional participants discussed in detail how to provide feedback of patient-reported SDM scores to clinical teams, evaluating the acceptability of different approaches to using feedback. Interviewees initially discussed surgeons receiving feedback as personal notification (email or text message) about poor patient-reported SDM experiences to prompt further intervention. This was found acceptable in case of a low number of patients requiring attention and sometimes preferred by some participants.

[T]he actual practicalities of how I want to be fed back, I wouldn’t be averse to having an email alert to say that somebody that’s under my care is worried about their surgery. […] I know we have a lot of emails, but if we had… I think because the numbers would be relatively low and we want to obviously nip those sort of issues in the bud, I think it’s a reasonable (HCP010)

I wouldn’t want it [feedback] to go anywhere else first. Especially if I’ve listed the patient and I’ve seen the patient, then it’s feedback to me really and I need to explore that further. (HCP013)

There was overall strong support for a process where feedback is initially provided to someone other than the surgeon. Professional participants frequently discussed the need for care pathway changes whereby introducing a “*filter*” (HCP011), a “*triage kind of mechanism*” (HCP010) or “*bringing someone in the middle*” (HCP003).

I think having a process delivered by someone who’s not the consultant as a first step would be more efficient and reliable. (HCP004)

Interviewees cited several reasons for the acceptability of this solution. Patients may prefer speaking to other staff due to communication issues or weak relationships with surgeons, and surgeons may miss feedback due to absence or competing demands. Participants also felt that other professional roles could often better address issues not requiring clinical input. While there was no consensus on who should receive feedback, professional participants commonly suggested specialist nurses or administrative staff (eg, waiting list coordinators, secretaries) in settings without them.

Another one that they might like to talk to a different doctor or a specialist nurse or some other member of staff because they didn’t get along with that particular doctor. (HCP008)

[M]y ideal would be one of your team, or one of my team has spoken to the patient and explored it, and said actually, these were the concerns, because then I can address them. (HCP005)

[Y]ou could weed out people that have just either made a mistake with the survey and got their scale the wrong way round. (HPC004)

I wouldn’t be averse to having an email alert to say that somebody that’s under my care is worried about their surgery […] because obviously I’d want to act on it. […] I suppose we need to think about what happens if the person receiving that email isn’t around and […] what the sort of process is to have a backup plan in terms of our maybe admin team. […] I suppose sort of looking at it in a sort of similar way to how we manage our kind of complaints, is that essentially the default is that they’re all for our admin managerial team to look at in the first instance and only come to us with the clinical stuff that can’t be dealt with by them. (HCP010)

#### Approaches to responding to feedback

Both stakeholder groups frequently talked about the need to refine processes for responding to feedback. All participants indicated that a process which provided a follow-up encounter (eg, another consultation discussion) would be acceptable to them to address deficiencies in patient-reported SDM experiences. There was emphasis from both participant groups that follow-up encounters should be tailored to the individual and address the specific issue underlying the poor SDM experience.

I want that opportunity to be able to discuss exactly what’s involved and you know if that can happen as a result of this survey then that’s great. (PAT003)

I would assume that the next step would be a follow up consultation to go through and make sure that the things that mattered most were discussed if they didn’t feel they had been already. (HCP013)

Most frequently, participants in both stakeholder groups thought that telephone conversations would be an appropriate and *“more convenient”* (HCP004) method for follow-up encounters. Some interviewees expressed a preference for face-to-face or virtual conversations because *“you can get a lot more information across”* (PAT006) but acknowledged the associated resource implications.

While some patient participants preferred to speak to a surgeon, most felt that additional communication can be with *“anyone from that department really that has the authority to be able to answer those questions”* (PAT003). Most often, patients mentioned nurses or specialist nurses as appropriate roles to address SDM deficiencies.

I think it’s a bit much to expect to speak to the surgeon, I know that’s what they're there for, but you don't want to take them away from what they’re meant to be doing. But it would have been nice to just have said, ‘If you want talk about your op…’ even if it had been 10 minutes, just to explain to me about exactly what I’m having done. (PAT017)

I feel that if I had put that survey score back in, but I still didn’t feel that I had the opportunity to discuss why and if somebody had have contacted me immediately and then said look is there anything we can do, it could have even been with the Breast Care Nurse, a meeting to discuss the specific concerns because I think that would have helped me, just even her acting as a conduit between the Consultant who didn’t have much time, but it would have only been a short discussion really. (PAT010)

Improving SDM experiences during follow-up encounters was widely viewed as essential to ensuring the credibility and effectiveness of real-time monitoring and feedback. Both participant groups felt that HCPs should lead this improvement by adapting their communication styles, with some patients also noting the need to be better prepared for treatment discussions.

I think maybe there’s somebody available, where you can just say, ‘Can I have a quick chat and ask more questions?’ because you go to your appointment, you’re not really prepared, and maybe I should have had questions, but I didn't at the time (PAT017)

I’m trying to understand what parts of the conversation are difficult to understand. Which parts are relatively straightforward to understand, and indeed which parts may need to be presented in a different way? (HCP009)

Successful engagement of staff in responding to feedback was a key concern mentioned by professional participants. Sharing data demonstrating current practices, for example, was identified as important for encouraging their involvement in follow-up encounters.

I mean it’s an extra step isn’t it? So I think if you’ve not engaged everybody into the process then getting people to act on the results might initially be difficult but if there’s a consistent trend, like I said, the reality is the data speaks for itself. (HCP002)

### Impact of real-time monitoring and feedback

#### Perceived impact on patients

Positive patient-level impacts of real-time SDM monitoring and feedback were commonly discussed by patient participants and by about half of professional participants. Both participant groups emphasised its value in realising patient-centred care.

what’s important for one person is really irrelevant to somebody else and unless you ask them what’s important for them, you don’t know (PAT010)

I think it’s incredibly important, the project [real-time monitoring and feedback of SDM]. I admire it because it is patient focused. Often, we tend to be process focused rather than patient focused, and this will just provide that additional insight into the patients experience that actually can modify things that sometimes we tend to ignore (HCP009)

Patient participants often felt that monitoring and feedback of SDM would positively impact their general satisfaction and happiness. Some interviewees emphasised how improved SDM may provide reassurance about their treatment decision and thereby decrease their worry about having surgery.

Just to get some clearer explanation as to you know, what exactly the surgery entails, somebody to listen to my concerns just so that I feel happier overall. […] I think the impact is you know, reassurance and peace of mind. I would just feel a lot more comfortable knowing what is going to happen to me than going in blindly putting literally my life in the hands of another person. (PAT003).

Similarly, professional participants mentioned increased satisfaction and improved treatment outcomes as potentially positive impacts.

[I]f the patient feels they’ve made an active decision with you, they will be much happier with the treatment outcome, whatever that is. (HCP007)

#### Perceived impact on surgeons

Potential impacts on surgeons were mentioned by both participant groups.

A few patient participants stated that real-time monitoring and feedback of SDM could help surgeons identify training needs or areas where they could provide better support.

It will help the surgeons then, they’ll think, ‘Oh, hang on I’ve got to help out a bit more,’ (PAT016)

I think they should learn from it and teach the solution, do you know what I mean? Not my individual solution but I’m sure [there are] courses and procedures where you have to go by what’s been said and how to deal with it. (PAT002)

Professionals often discussed positive as well as negative impacts of real-time SDM monitoring and feedback on their own practice. Interviewees also perceived real-time monitoring and feedback of SDM as a crucial part of their professional development and highlighted they would *“be open to learning from that sort of data”* (HCP010). Specifically, the majority felt that receiving information about patients’ experience with SDM was an opportunity for reflection and improvement in communication skills. Interviewees were interested in personal performance trends and felt there was value in receiving analytical feedback from aggregate scores over time.

I think that the utility of this tool is in providing aggregate feedback. (HCP011)

My view is, if you get negative feedback, that’s much more important than any positive feedback because somebody’s really wanted to say that, if you see what I mean. They’ve really felt—they’ve climbed and crossed a threshold in order to actually criticise somebody who’s trying to help them. So, from my point of view, certainly that negative side, those themes that might come out on an analysis over a period of time were probably more helpful than the individual cases.[…] it’s giving the clinician the tool to examine their general practice so that they can modify and improve matters. (HCP009)

However, challenges with regard to individual surgeons’ motivation to change and improve their communication style were also acknowledged.

I guess you’ve got to be wanting to improve before with this information, rather than thinking: ‘Gosh, well I do a good enough job anyway and I don’t care what anybody says’. But there will be people like that but there’s nothing you can do about that (HCP009)

I’d be interested in knowing is whether they can actually change their communication style to the extent… If you feed this back to a 55 year old surgeon, can that person actually change their style in the last few years of their career, to improve their patient satisfaction? I don't know. (HCP007)

Use of SDM data for appraisal and revalidation was sometimes discussed by professionals. Participants were divided over whether the data should be used for formal performance evaluation. Some interviewees perceived it as “a much better way of doing it [revalidation]” (HPC008), whereas others were sceptical, expressing concerns about the potential misuse of SDM measurement for performance management and the overlap with existing performance reviews.

I suppose the only thing is every time a Trust measure something there’s a risk that they’re using it for performance management […] and is that a bad thing or is it a good thing? (HCP004)

I think the difficulty is whether it is going to end up being part of your revalidation I guess is something that might be considered. Yes, I think that might be one way to take it and then I guess to show reflection and if you have changed your style (HCP012)

We already get a lot of reviews of our performance from a cancer perspective and it [the national prostate cancer audit] already surveys the quality of information (HCP002)

Interviewees were often concerned about possible additional burden resulting from receiving negative feedback about patients’ SDM experience. Some expressed worries about increased workload to address feedback which was often discussed in connection with existing performance pressures (eg, reviews on cancer targets). Others viewed actions on feedback as manageable if these are offset with a reduction in complaints in the long term.

I know it’s only a phone call, but it is another phone call – it all adds burden, time (HCP005)

The tricky bit is going to be if your patient thinks that they’ve been hard done by and you’ve got to try and meet their cancer target, you’re suddenly going to go ‘how am I going to fit all that in before their operation in a week’s time? (HCP003)

I would feel completely happy with that, assuming I’m not going to get 40. […] We get feedback in lots of different ways already and again, if it can stop complaints because I think what you are trying to do is almost pre-empt that. (HCP012)

Professional participants occasionally highlighted emotional burden as a negative consequence of receiving negative feedback.

I pride myself on my communication and if someone said […] that I have made no attempt to… and that is pretty awful. Of course I’d be upset about that (HCP004)

#### Perceived impact on departments or the organisation

Organisation or department-level impacts of SDM monitoring and feedback were rarely mentioned by patient participants but were discussed by all professional participants. These commonly included the potential to detect and address wider systemic performance issues.

Well, it’s making sure that any errors or problems that there might be with the system, or the procedures that are in place, any problems that they might have can be identified and eliminated. (PAT006)

I suspect that might be an area where we don’t do so well. […] I think if there are particular themes within our department then we could look to address that (HCP012)

Professionals further discussed the potential for larger-scale training in connection with the importance of monitoring trends and analysing cumulative data. For around half of the interviewees, the benefit of cohort-level feedback outweighed the benefits of receiving feedback on an individual, patient-level. This extended to the desire to “see how we ranked vs other specialities, other clinicians, you know anonymised” (HCP008).

It would be incredibly useful to have a sort of annual appraisal style report to allow you to influence practice, and I think it would be extremely helpful to flag up when there are any trends. (HCP005)

Professional participants sometimes pointed out wider impacts such as reputational benefits for the organisation resulting from potentially reduced complaints and litigation, or the value of gathering evidence to inform improvements to hospital processes (eg, longer clinic times).

I mean, I think from a hospital perspective and an organisation perspective, maybe the hope is that they might, you know, the great thing about this is it might avoid people complaining. (HCP002)

Something we talk about fairly regularly is about whether we should be doing a consent clinic for the patients we’re operating on in two weeks’ time and there is so much pressure on clinic spaces and our time that we haven’t done it, you know. I suppose we were surveyed and we were scoring low and additional phone calls weren’t solving it then you can say, “Well, actually, we’ll formalise this and everyone who’s having an operation in the next two weeks is booked, schedule two hours for phone calls for patients next week. (HCP006)

## Discussion

This qualitative study explored the acceptability and impact of real-time monitoring and feedback of patients’ experiences of SDM measurement. 10 themes were identified describing the perspectives of surgical patients and HCPs in relation to acceptability of real-time SDM monitoring, real-time feedback and addressing SDM deficiencies and impact on clinical practice. Findings suggest that integrating an automated system for real-time SDM measurement and feedback within surgical pathways of NHS Trusts is acceptable. It was endorsed by both groups who also felt it would be valuable to improve the process of care where deficiencies were identified. Results emphasised the complexities of SDM measurement in surgical contexts, specifically regarding the timing of the SDM monitoring, how and what feedback was implemented and actioned within the care pathways, and the differing SDM needs of individuals with various conditions. These complexities might significantly impact the acceptability of real-time monitoring and feedback, with some issues being of particular importance to patients (eg, limited inclusivity of real-time SDM monitoring) and others unique to professionals (eg, the need for additional qualitative feedback alongside SDM scores). Insights also revealed support for real-time SDM monitoring and feedback because of the potential to improve patient-centred care and associated benefits to patients, surgeons and clinical teams, and the wider organisation. Facilitators and barriers at all levels were identified that can affect the adoption of future interventions aimed at improving SDM.

### Implications for research

The acceptability and impact of real-time electronic measurement and feedback have been explored in other healthcare contexts which examined other types of patient-reported data, such as symptoms and health-related quality of life scores,^31 43^ clinical outcomes^44^ or safety.^45^ Findings in those studies align with results reported here, demonstrating that clearly presented feedback on patient-reported outcomes and user-friendly technology can enable adoption of similar systems. To our knowledge, however, no previous research has yet investigated stakeholders’ views towards integrating, at scale within a healthcare setting, the real-time monitoring and feedback of patients’ experiences of SDM. This study provides an important first step to addressing the UK National Institute for Health and Care Excellence call for research into new interventions targeting inclusive, sustained, health system-level SDM improvement.^14^ Results add to our understanding of how to efficiently and routinely monitor patients’ SDM experiences at scale, and it adds insights into factors impacting the acceptability among key stakeholders that are necessary to inform intervention development for organisation-wide improvements in SDM.

Insights gained from this study are crucial to inform the development, implementation and evaluation of an SDM improvement intervention in alignment with the principles of the Medical Research Council’s framework for the development and evaluation of complex interventions and the Health Technology Assessment framework.^33 46^ Factors were identified relevant to optimising the acceptability of a system for real-time SDM monitoring and feedback that can be used to inform the codesign of components of a future SDM intervention that meet the specific needs of key stakeholders. For example, identified themes suggest that the approach to providing feedback of SDM scores to clinical teams will require careful management along the pathway of care. Participants highlighted consequences of potential emotional burden and disengagement in cases of negative feedback. Patient and professional-facing communication may therefore consider strategies linked to positive message framing to optimise engagement with SDM monitoring and feedback. Concerns regarding the reliability and validity of SDM data were prominent in this study, affecting acceptability of routine SDM monitoring and feedback in particular among professionals. These concerns reflect ongoing debates in the field where significant challenges in SDM measurement quality have been well documented.^12 47 48^ Many existing instruments lack high-quality psychometric and methodological foundations,^49^ and studies have highlighted misalignment between patient, provider and observer assessment of SDM quality.^50 51^ Such discrepancies raise important questions about conceptual deficits and valid perspectives in SDM measurement that warrant attention in future research.

SDM is widely recognised as a multifaceted process with various conceptual models describing the steps to be taken, or core elements necessary to achieve high-quality SDM.^52^ Importantly, SDM can be an ongoing process and ‘distributed over time, place and between different participants’.^53^ Participants’ narratives in this study reflected this complexity, describing multiple interactions with different healthcare providers, including primary care and perioperative services. Interactions during any of these encounters are likely to contribute to the collective process of decision-making and affect patients’ overall SDM experience.^54^ Distributed decision-making has also shown to exclude patients from involvement in decision-making.^55^ These dynamics present challenges for capturing patient-reported SDM experience at a single, fixed time point and underscore existing research gaps regarding the optimal timing of SDM measurement.^12 48^ Routine SDM measurement following a surgical consultation may only represent a narrow snapshot or would give little insight into deficiencies that arose earlier in the SDM process.^56^ Addressing this limitation may require a deeper understanding of individual patient pathways and personal contexts, as well as more collaborative, longitudinal approaches to SDM assessment to identify how patients develop informed preferences. There is, however, a lack of evidence to understand how SDM can be effectively supported across varying time, places and participants. Some SDM models have taken distributed decision-making into account during their theoretical development^57^ and emphasise the need for practical guidance for clinicians to incorporate, for example, diverse information sources and patients’ social networks into the decision-making processes.^58 59^ Some more radical suggestions include restructuring consultations to include perspectives from a range of healthcare providers.^55^ There are important future research opportunities to explore how SDM measurement can be optimised and how SDM can be supported in the context of distributed decision-making in surgery.

The use of digital systems to monitor patient outcomes in surgery has accelerated since the COVID-19 pandemic, but the need to prioritise health equity and research to improve language and digital inclusivity is widely acknowledged.^60^ A related study has found a range of factors affecting use and engagement with the system for real-time SDM monitoring.^29^ Patients particularly valued its low burden and ease of use, noting that the clear, accessible design supported participation across a diverse range of users, including those with varying levels of health literacy and digital confidence. This study explored the views of additional key stakeholders and reports insights gained above and beyond other related work. Participants highlighted the potential for effectively reaching a large number of patients and the ability to adapt the system to suit different populations of patients and different contexts. More work is needed, however, to inform intervention design which supports inclusive, sustained, health system-level improvements in SDM. A complementary study (in preparation) examined the views of members of the public representing underserved groups (ie, elderly, economically disadvantaged and ethnic minority) on real-time SDM monitoring and feedback.^28^ Specifically, this work used non-English, face-to-face qualitative data collection drawing on citizen science approaches^61^ to explore and understand how inclusivity can be optimised in this context. For example, it is anticipated that the decision support intervention will include non-English and non-digital components, and integration of services that proactively reach under-represented populations. Specifically, findings suggested translations of SDM measurement instruments, using interpreters and supported completion of the electronic system (eg, on tablet computers in clinic, supported by staff/family) to meet inclusivity and accessibility needs and maximise engagement. In addition, it is important to explore the views of other relevant professions (eg, NHS managers, administrative staff) in future research to understand other relevant system-wide barriers and requirements to the successful implementation of a system for real-time monitoring and feedback. Planned future intervention development work will incorporate views of a wider and more diverse range of professional stakeholders and communities to address relevant factors identified through formative work.

### Strengths and limitations of this study

Strengths included use of robust qualitative methods which explored and compared perspectives from both patients and HCPs. Investigating perspectives of key stakeholders who had the opportunity to engage with the electronic system obtained new insights about important issues impacting acceptance and implementation of a real-time SDM monitoring and feedback. The study was conducted in a real-world clinical setting and will likely inform wider implementation of the system in UK NHS Trusts.

Limitations include a self-selected sample that lacked diversity. Consideration of ethnicity, diversity and inclusion principles is particularly important in the design and development of digital health interventions^62^ and for improving SDM.^63^ Recruitment of patient participants from a more diverse range of population groups was hindered by a lack of comprehensive ethnicity data in the hospital electronic patient record system. This meant that ethnicity data were not used as an a priori sampling criterion but collected retrospectively. Similarly, governance approvals restricted the eligibility criteria to patients who have already completed SDM monitoring. It may be that patient participants in this study were comfortable with using digital technologies and interested in enhancing patient involvement in their care. Likewise, the self-selection of professional interviewees poses a further bias. HCPs represented three of seven surgical specialties. Recruitment relied on convenience methods and depended on surgeons’ availability and willingness to participate in interviews. While participation from all surgeons within departments was actively encouraged, including those who might hold opposing views, it is possible that individuals less interested in improving SDM or those constrained by heavy workloads lacked the motivation or capacity to take part. These limitations may help to explain why themes frequently reflected positive perspectives of the system and might mean additional critical viewpoints were not captured.

### Clinical implications

International health policy emphasises the importance of high-quality SDM processes in patient-centred care.^2 9 10^ Results have direct relevance for health service or quality improvement managers seeking to enhance SDM in practice. Implementing real-time monitoring and feedback of SDM addresses a key policy recommendation to track SDM at large scale.^12^ It can, therefore, be a critical mechanism to guiding new interventions for targeted, sustained improvement activities at organisation level.

Sustained improvements in SDM, however, require complex interventions involving multiple stakeholders at different levels of the organisation.^11 15 33 64^ Although the findings identified several factors influencing the acceptability of real-time SDM monitoring and feedback within clinical practice, there are many unknowns as to what components and mechanisms are needed for all stakeholders to ensure its effective adoption and impact on service outcomes and patient benefit. Research to formally develop and evaluate a decision-support intervention using real-time SDM monitoring and feedback is needed to improve the evidence base. Feedback components, for example, have been widely used to prompt enhancements in professional practice, but the mechanisms through which consistent improvements can be achieved are not well understood.^18 27^ Findings highlighted real-time SDM monitoring and feedback provided opportunities for HCP reflection, potentially prompting practice improvements. This can be crucial for enhancing clinical reasoning that underpins high-quality SDM^65^ and may be a key mechanism through which decision support interventions help achieve higher standards of care in this field. Future intervention development work should draw on specific theories (eg, Clinical Performance Feedback Intervention Theory^27^) to help explain the specific factors that influence feedback success to enhance the effectiveness of an intervention that uses real-time SDM monitoring and feedback.

Findings suggest that the implementation of real-time SDM monitoring and feedback is a service-wide responsibility. Results highlighted the need for careful consideration of core responsibilities and complexities when remediating patient-reported deficiencies in SDM. Both professional and patient participants noted that surgeons are not always the most appropriate to lead SDM improvements and discussed the potential suitability of other members of the clinical or departmental team members (eg, nurses, administrative staff) in certain contexts. Careful consideration should, therefore, be given to identifying appropriate professional roles for receiving feedback and defining new processes for how services should respond.^66^,^70^ Results highlight the need to codesign approaches to ensure tailored responses to SDM feedback are acceptable to patients and professionals. This includes identifying suitable roles within each surgical department, defining new responsibilities collaboratively with teams and co-developing supportive materials (eg, new standard operating procedures) to ensure seamless integration into existing workflows. Substantial challenges to widely adopting SDM in clinical practice, however, have been noted,^66 67 71^ and various facilitators and barriers to implementation have been identified.^68^,^70^ Any programmes that aim to routinely embed SDM monitoring and feedback should build on existing knowledge about mechanisms that support the adoption of SDM in practice^15 72 73^ and may use strategies for embedding SDM into care pathways at varying levels, including micro (individual), meso (departmental) and macro (organisational) levels.^65^ Established frameworks from behavioural and implementation sciences (eg, theoretical domains framework, normalisation process theory^74 75^) could help in developing approaches systematically and transparently which incorporate relevant stakeholder insights and map context-specific barriers and facilitators. Additionally, more work is needed to develop effective strategies for stakeholder engagement that can address identified HCP concerns (eg, worries about time pressures, performance evaluation or scrutiny) to maximise uptake of the future intervention to improve SDM.

## Conclusions

This study explored patient and HCP views towards a novel system for automated, real-time monitoring and feedback of surgical patients’ experiences of SDM integrated within elective surgical care in two English NHS Trusts. The system was acceptable to the sampled population and well-positioned to form the basis for future intervention development to improve SDM. Ongoing work addresses the need to understand views of a more diverse sample to ensure the acceptability of SDM to the general UK population. Findings will guide effective strategies for wider implementation of real-time SDM monitoring and feedback in the NHS.

## Supplementary material

10.1136/bmjopen-2025-099090 online supplemental file 1

## Data Availability

Data are available on reasonable request.
