# Supplementary material for: Patient and surgeon perspectives of a large-scale system for automated, real-time monitoring and feedback of shared decision-making integrated into surgical practice: a qualitative study
Source: BMJ Open. 2025 Jun 27;15(6):e099090. doi: 10.1136/bmjopen-2025-099090 (PMC12207162; doi:10.1136/bmjopen-2025-099090)
Supplement: online supplemental file 1 [file bmjopen-15-6-s001.docx]

**ALPACA - Topic Guide: Patient participants**

| **Background** | - Intro to interviewee (name, role and inability to answer care-related questions) - Explain research project different to hospital - Reminder of anonymity, confidentiality and that interview can be stopped at any time.   Thank you for taking the time to talk to me about your experiences with decision making at Southmead Hospital. As part of the project, we are speaking to patients to try and support improvements to patients experience with SDM. So, it is really valuable for us to understand your personal experience so we can improve things for future patients.  Before we start, do you have any questions about the study or the information that were sent to you?  Can I also check again that are you happy for our conversation to be recorded?   - *Switch on recorder* | |
| --- | --- | --- |
| **Warm up and explore pathway to-date** | - Explore if participant is scheduled for operation/had an operation/talked about surgical treatment with a consultant.  1. Can you tell me a little bit more about what the surgery involves/conversation with the consultant involved? | |
| **Exploring patients’ views on:**   - Personal scores | 1. What were your initial thoughts when receiving the survey?   I am particularly interested in the first 3 questions you have completed [*Read out questions if needed*]   1. Could you tell me a bit more about how you have responded to the survey? 2. Why did you answer the questions in this way? | - Can you explain why you thought/felt *XYZ*? |
|  |  | - Why did you score question 1, 2, 3 in that way? - What differences did you see between the questions? |
| - Feedback and actions | Thanks for explaining this to me in more detail. Now that you have had this bad experience…   1. How can/could have the hospital make things better? | - What else could be done so you feel you had a better experience with decision making? *Why*?   The hospital is going to ask you these questions again the day before surgery.   - What else would the hospital need to do so that your scores go up? *Why?* |
|  | 1. What aspects of your experience should be fed back to the surgical team? *Why? And how?*   OR   1. What do you expect happens next? | - How/Why do you think this would improve your experience? |
|  | 1. How do/did you feel about going ahead with the planned surgery? | - Would/did you consider delaying or cancelling surgery until your experience has improved? |
| - Impact | Thank you for sharing your experience, that is really helpful for us to improve how we go about addressing bad experiences in future. I am also interested to hear how you generally feel about the hospital wanting to improve patients’ experience with decision making?   1. How do you feel about the hospital asking about your decision-making experience using a survey? *Why*? | - Which aspects do you think are good/bad? *Why*? - What impact might this have? *Why*? |
| - Acceptability | 1. What do you think about the hospital using an online survey (text/email) to capture peoples experience with decision making? | - What concerns do you have? *Why*? - Why do you think some people would not complete this survey? *Why*? |
| **Wrap up** | 1. I’d be interested to know how else you think the hospital should use the information you provided? 2. Finally, would you like me to pass on any of the feedback you shared with me to the clinical team so they can get in touch with you? If so, what information would you like me to share?  - *Wrap up interview* - Ask whether participant is happy to be contacted again for a second interview if necessary (agree on acceptable mode of contact) - *Thank participant* | |

**Alpaca Topic Guide: Professional participants**

| **Background and warm-up** | - Intro to study (if needed) and interviewer (*gauge PTs previous knowledge of QI project*)   - Improvements in SDM through real-time monitoring of patients experience (via ePROM) and feedback of their scores   - Share CollaboRATE questionnaire and explain ePROM   - Purpose of interview: how to design feedback, which patients we should notify you about, what actions you would take - Reminder of anonymity and check if audio recording is ok - Any questions? | |
| --- | --- | --- |
|  | Imagine you have just completed a consultation discussion with one of your patients, a treatment decision has been made and the patient gets booked in for surgery. Two days later, you receive an email notifying you that they had a poor experience with shared decision making. So, imagine you have just received the email and are about to open it… | |
|  | **Questions** | **Prompts** |
| - Content, email 1 | 1. What would you expect to see in this email alert? *Why?* | - What else would you like to see? *Why?* |
|  | 1. What do you think should be the key message or feature in this email to make you want to action on the email alert? *Why?* | - What else do you think is an essential bit of information that would prompt you to action? *Why?* |
| - Relevance of existing content | 1. How would you feel if the email included:    1. The overall score that triggered the alert    2. The scores per question    3. Median/IQR scores of other patients    4. A link to view the relevant patient record | - Why do you think XYZ is relevant/not relevant? |
| - Threshold | Please look at the collaborate scores (appendix 1)   1. How do you think we should decide on a threshold? *Why?* | - What patients would you like to be notified about? |
| - Remedial action | Going back to the scenario then, let’s say you have just opened such an email and it is alerting you about the patients’ bad experience with SDM…   1. What action do you think you would take to try and shift the patients’ responses? *Why?* | - What else would you do? *Why?* - *Show questions (appendix 2)* |
|  | 1. How do you feel about an intermediary between you and the patient? | - Who are the people you would want to intervene following the intermediary? |
| - Acceptability | Thanks a lot for your suggestions. We have talked a lot about the feedback and actions, but I am also interested in your views on the project in general …   1. How do you feel about the hospital wanting to improve SDM in this way? *Why?* | - What aspects are good in particular? - Which aspects worry you? Where do you see the challenges? |
|  | 1. What do you think about using the survey as a means for real-time measurement of SDM? | - What are the negative/positive aspects? - How can we address XYZ? - What can we change about the project? |
| - Impact | 1. What impact do you feel will the project have? | - What difference do you think it will make? - Who do you think will benefit most from this project? *In what way?* - What are the consequences? *Why is this important?* |
| **Wrap up** | 1. Finally, I’d be interested to know what YOU think how the hospital should use all the information collected? *Why?*  - *Wrap up the interview* - *Can you think of any colleagues who we could interview about this?* - *Thank participant and reminder of observations when receiving the actual feedback and follow-up interview* | |
