# Supplementary material for: Patient and surgeon perspectives of a large-scale system for automated, real-time monitoring and feedback of shared decision-making integrated into surgical practice: a qualitative study
Source: BMJ Open. 2025 Jun 27;15(6):e099090. doi: 10.1136/bmjopen-2025-099090 (PMC12207162; doi:10.1136/bmjopen-2025-099090)
Supplement: Supplementary file 2 [file bmjopen-2025-099090.reviewer_comments.pdf]

# PEER REVIEW HISTORY

BMJ Open publishes all reviews undertaken for accepted manuscripts. Reviewers are asked to complete a checklist review form and are provided with free text boxes to elaborate on their assessment. These free text comments are reproduced below.

**ARTICLE DETAILS**

**Title (Provisional)**

Patient and surgeon perspectives of a large-scale system for automated, real-time monitoring and feedback of shared decision-making integrated into surgical practice: a qualitative study

**Authors**

Hoffmann, Christin; Avery, Kerry; Macefield, Rhiannon; Snelgrove, Val; Rooshenas, Leila; Bekker, Hilary; Hopkins, Della; Cabral, Christie; Blazeby, Jane; Gibbison, Ben; Hickey, Shireen; Williams, Adam; Aning, Jon; Judge, Andrew; Smith, Andrew; Lingampalli, Archana; Reeves, Barnaby; Preshaw, Jessica; Whitehouse, Michael R; Cresswell, Paul; Braude, Philip; Potter, Shelley; Beckitt, Timothy; Whittlestone, Timothy; McNair, Angus GK

---

**VERSION 1 - REVIEW**

---

| | |
|---|---|
| **Reviewer** | **1** |
| **Name** | **Pieterse, Arwen** |
| **Affiliation** | **Leiden University Medical Center, Biomedical Data Sciences** |
| **Date** | **15-Feb-2025** |
| **COI** | **None** |

---

Thank you for the opportunity to review this manuscript describing a qualitative investigation of the acceptability and perceived impact of a large-scale system integrated in several surgical pathways, to provide automated feedback on shared decision making. The manuscript reads well and is an important step in the further development of this intervention. Please find my comments detailed below.

1. P7, line 17: The authors seem to posit shared decision-making (SDM) as something 'good' for healthcare decision making, such as about surgery (eg, "Health policy recommends a comprehensive approach to improving SDM in practice."). It comes across as if SDM should be attempted with any decision about surgical treatment, regardless of the patient's particular condition and possible options. Is this indeed the intention of the authors to

convey this message? If so, please substantiate that position explicitly. If not, then rephrase to make clear when SDM is relevant.

2. P7, line 28-35: I am wondering about the rationale presented here. To what extent can feedback on patient-reported symptom and its effect on patient survival be compared to feedback on patient-reported SDM and SDM with other patients? I wonder if the authors could make their reasoning explicit. In the former case, care can be aligned better to the patient's actual condition. In the latter case, it presumes that receiving feedback on how a patient experienced the interaction is useful (or enough) for clinical teams to adapt their communication such that SDM improves with other patients. Not only the patients are different, the process of SDM is also something the healthcare professional and patient do together. What evidence is there, that this improvement based on feedback is indeed expected?

3. P8, line 48: The term 'monitoring' does not clearly seem to cover what it means, namely, the routine collection of patient-reported data. Monitoring suggests as if real-time intervention is possible if the process does not go as desired. This (implicit) meaning also transpires through the description as used on p15, line 16-18 ("It was widely recognised that monitoring (i.e., the survey) has the potential to detect SDM deficiencies and provides opportunities to improve SDM experiences.") - even though the collection of data in itself does reveal anything. The authors may wish to consider choosing a descriptor that reflects the activity in a more transparent manner.

4. In their protocol paper, the authors indicate that they have a particular interest in underserved patient groups in the co-creation of their intervention. They mention economic disadvantage, older age, and being a member of an ethnic minority as relevant characteristics in that respect. In the present manuscript, they recorded age and sex (p9, line 50-54), and have not recorded patient economic status or ethnicity. The authors could not therefore, by design, purposively sample on relevant characteristics, to evaluate the acceptability and impact of the intervention. Please explain this choice.

5. In relation to the point above: the authors state that they did not record participant ethnicity, yet report that all patient participants were " from a white British background". Please clarify (p12, line 45).

6. Table 2: The summaries describing the themes "Complexities of SDM measurement" and "Challenges of distributed decision making" seem to overlap, both refer to how hard it may be to interpret SDM results. The same holds for the summaries describing the themes 'Approaches to responding to feedback' and 'Approaches to providing feedback to clinical teams'. Please make distinction more clear, or collapse themes. Note that in both cases, the distinction between the themes is clear in the text (paragraphs 1.2-1.3, 2.1-2.2).

7. P21, lines 48-52: The quote by PAT017 is telling about how this patient (and maybe others too) view as the core task of a surgeon: "I think it's a bit much to expect to speak to the

surgeon, I know that's what they're there for, but you don't want to take them away from what they're meant to be doing. […].” (PAT017)

This is an essential point with regard to the whole endeavor of trying to improve SDM in this setting: to what extent is there buy-in both from patients and healthcare professionals, that SDM is worth improving since it may be what they (patient together with the surgeon) are meant to be doing. If not, what then is the point of providing this feedback to surgeons? I invite the authors to consider to what extent similar ideas about roles were expressed by their participants and if the implications may be worth discussing.

8. P26, line 28-32: The authors state that “This study provides an important first step to addressing the UK National Institute for Health and are Excellence call for research into new interventions targeting inclusive, sustained, health system-level SDM improvement [10].” However, as they have noted before, their real-time SDM monitoring was perceived to be limited in its inclusivity, an issue of particular importance to patient. Also, they note a lack of diversity in their sample, in terms e.g. of ethnicity (although this was stated not to have been measured), including only patients who had completed SDM monitoring, using convenience sample of healthcare professionals, and distributing only an English version of the SDM-survey. Please provide an explicit rationale for why the present system nonetheless may be inclusive.

Textual:

P 7, line 8-9. It seems a bit odd to use the Cochrane review by Légaré et al (2018) as evidence to support that SDM can impact health outcomes. Please review and adapt.

P9, line 38-39: The third eclusion criterion is unclear, please rephrase.

P9, line 40-41: It is unclear what “those” refer to in “A sub-set of those patients were sampled…], since immediately before the authors have described which patients have been excluded.

P9, line 50-52: What do the authors mean by “Participant characteristics were assessed as the study

progressed and recruitment efforts targeted under-represented patients.”? Please clarify.

P10, line 12-16: The sentence “This involved multi-disciplinary … more details) [31–33].” Does not read fully well, please correct.

P21, line 20-21: What is the distinction between “conversation” and “consultation encounter”? Please explain.

---


| | |
|---|---|
| **Reviewer** | **2** |
| **Name** | **Clapp, Justin** |
| **Affiliation** | **University of Pennsylvania** |

| | |
|---|---|
| **Date** | **19-Mar-2025** |
| **COI** | **None** |

Thanks for the opportunity to review this manuscript. The authors report a qualitative interview study of patients and surgeons about a system for real-time patient reporting on shared decision making (SDM). The manuscript is clearly written, the methods are comprehensively laid out, and the results are well organized and supported nicely with many illustrative quotes. The sample characteristics aren't perfect but the authors appropriately acknowledge this in the limitations.

I have one major piece of feedback. The results are quite long for a clinical journal—to the point where I'd strongly consider slimming them down to the themes the authors find most important/impactful. Meanwhile, I didn't find the discussion to offer a terribly robust engagement with the findings. I'd suggest extending the discussion and shortening the results. The interviews have revealed some issues that have quite interesting implications for the attempt to characterize SDM and report it to practitioners. For example, the surgeons questioned whether patient-reported experiences actually reflect the quality of surgeons' communication. They have a good point here: tools for the measurement of SDM (observational and patient-reported) have not been found to have good correspondence in several studies. In my experience doing ethnographic work combining interviewing and observation, I've similarly found that the degree to which patients describe being involved in a consultation often is not apparent in the consultation itself. The points that patient and surgeon interviewees made about the distributed nature of decision making also have major implications for any effort to characterize and report SDM. Since in many areas of medicine care is increasingly done via multidisciplinary teams, entailing chains of clinical interactions, it's possible that various elements of SDM are being accomplished at various stages. I'd like to see the authors address these kinds of issues at greater length in the discussion, engaging with relevant literature; I think it'd substantially enrich the manuscript.

## VERSION 1 - AUTHOR RESPONSE

**Response to reviewers**

**Reviewer: 1**
Dr. Arwen Pieterse, Leiden University Medical Center
Comments to the Author:
Thank you for the opportunity to review this manuscript describing a qualitative investigation of the acceptability and perceived impact of a large-scale system integrated in several surgical pathways, to provide automated feedback on shared decision making. The manuscript reads well and is an important step in the further development of this intervention. Please find my comments detailed below.

We thank the reviewer for their careful reading and helpful suggestions. We have addressed all comments raised and believe that this has significantly strengthened the manuscript. Please find below out point-by-point response which highlight the specific changes made.

**COMMENT 1.1**

1. P7, line 17: The authors seem to posit shared decision-making (SDM) as something 'good' for healthcare decision making, such as about surgery (eg, "Health policy recommends a comprehensive approach to improving SDM in practice."). It comes across as if SDM should be attempted with any decision about surgical treatment, regardless of the patient's particular condition and possible options. Is this indeed the intention of the authors to convey this message? If so, please substantiate that position explicitly. If not, then rephrase to make clear when SDM is relevant.

**RESPONSE 1.1**

Thank you for suggesting to clarify the scope of our work. We agree that the aim to achieve high-quality shared decision making (SDM) may not be universally applicable or equally important to all surgical decisions. We made minor amendments to the introduction section to acknowledge this and have added a reference to support this.

Amended sections on pp 5, 7 now read: "High-quality SDM discussions for many surgical treatment decisions is particularly important, especially those that are preference-sensitive, because consequences are usually irreversible [1]."

The study explores the acceptability of a hospital-integrated SDM measurement system with an intentionally large scope and inclusion of a range of surgical procedures and decision contexts. This reflects the wider programme's aim to support organisation-level improvements in SDM. There are indeed some decision contexts excluded in this research programme where SDM may be less relevant. We have now included explicit detail about these in response to comment 1.10. In addition, we have also added detail in the Study context section to clarify the inclusion of a large scope of decision contexts. The corresponding section now reads:

*"This study was part of a larger programme of work (the ALPACA Study) that seeks to co-develop a decision support intervention to improve SDM practices within surgical pathways[2]. The intervention is expected to use a system for automated and real-time monitoring and feedback of patients' experiences of SDM before surgery. It is designed for broad integration across multiple surgical specialties to support organisation-level improvements in SDM by enabling scalable, routine SDM measurement across a large range of surgical decision contexts, including, for example, cancer or benign, and more or less preference-sensitive treatment decisions."*

**COMMENT 1.2**

2. P7, line 28-35: I am wondering about the rationale presented here. To what extent can feedback on patient-reported symptom and its effect on patient survival be compared to feedback on patient-reported SDM and SDM with other patients? I wonder if the authors could make their reasoning explicit. In the former case, care can be aligned better to the patient's actual condition. In the latter case, it presumes that receiving feedback on how a patient experienced the interaction is useful (or enough) for clinical teams to adapt their

communication such that SDM improves with other patients. Not only the patients are different, the process of SDM is also something the healthcare professional and patient do together. What evidence is there, that this improvement based on feedback is indeed expected?

**RESPONSE 1.2**

Thank you for raising these interesting points and we agree that further detail is needed to explain the role of feedback in improving in care and outcomes. We have significantly revised this section in the introduction which now reads:

*"In addition, feedback of patient-reported measures, either as key intervention component or on its own, is a widely used quality improvement intervention [18,19] and has shown to positively impact patient care and outcomes in other contexts [20–22]. For example, a trial evaluating integrated, real-time symptom monitoring with feedback to clinical teams has led to improvements in overall two-year survival in advanced cancer patients by enabling clinicians to respond timely to patient-reported symptoms and preventing downstream adverse complications [23]. Similar interventions in chronic disease patients improved outcomes due to early detection of problems [24]. Therefore, it is plausible that measuring patient experiences of SDM can detect deficiencies early and feedback to clinicians can be promising mechanisms to prompt efficient remediation of problems, improving SDM experiences before surgery. The optimal approach for feedback design and implementation, however, remains to be explored. A better understanding of key stakeholders perspectives can optimise the design of feedback mechanisms to drive meaningful and sustained behaviour change within clinical teams along the surgical care pathway [25–27]."*

**COMMENT 1.3**

3. P8, line 48: The term 'monitoring' does not clearly seem to cover what it means, namely, the routine collection of patient-reported data. Monitoring suggests as if real-time intervention is possible if the process does not go as desired. This (implicit) meaning also transpires through the description as used on p15, line 16-18 ("It was widely recognised that monitoring (i.e., the survey) has the potential to detect SDM deficiencies and provides opportunities to improve SDM experiences.") - even though the collection of data in itself does reveal anything. The authors may wish to consider choosing a descriptor that reflects the activity in a more transparent manner.

**RESPONSE 1.3**

The reviewer raises a good point and we agree with this observation that there are several components to the process of monitoring. We have added detail to our "Study context" section to clarify that by "monitoring", we mean the multi-step process: *"Incorporating routine data into clinical practice is a multistep process involving systematic measurement and data collection, analysis, interpretation and integration into clinical care. Our protocol describes this process as "SDM monitoring"*. We have carefully reviewed the manuscript and have ensured that the use of the word 'monitoring' aligns with the process stated above and that 'measurement' is only used when we mean the collection of SDM data through the electronic survey.

**COMMENT 1.4**

4. In their protocol paper, the authors indicate that they have a particular interest in underserved patient groups in the co-creation of their intervention. They mention economic disadvantage, older age, and being a member of an ethnic minority as relevant characteristics in that respect. In the present manuscript, they recorded age and sex (p9, line 50-54), and have not recorded patient economic status or ethnicity. The authors could not therefore, by design, purposively sample on relevant characteristics, to evaluate the acceptability and impact of the intervention. Please explain this choice.

**RESPONSE 1.4**

Many thanks for this observation and we acknowledge that further detail is needed to explain the different component studies of this programme of work. The study the reviewer is referring to constitutes a separate and complementary piece of work which is now completed and in preparation for submission. The present study represents the initial qualitative exploration with patients and professionals who have engaged with the electronic system. Our extensive PPIE work indicated that more substantive research activities are needed to understand how the electronic system for real-time monitoring and feedback can be made more inclusive of under-served groups. A separate study was therefore undertaken to investigate the views of elderly, ethnic minority and economic disadvantaged population groups and will be reported separately.

This was made clearer in the relevant discussion section, together with changes in response to comment 1.8. The amended section now reads:

*"This study explored the views of additional key stakeholders and reports insights gained above and beyond other related work. Participants highlighted the potential for effectively reaching a large number of patients and the ability to adapt the system to suit different populations of patients and different contexts. More work is needed, however, to inform intervention design which supports inclusive, sustained, health system-level improvements in SDM. A complementary study (in preparation) examined the views of members of the public representing under-served groups (i.e. elderly, economic disadvantaged and ethnic minority) on real-time SDM monitoring and feedback [2]. Specifically, this work used non-English, face-to-face qualitative data collection drawing on citizen science approaches [13] to explore and understand how inclusivity can be optimised in this context. For example, it is anticipated that the decision support intervention will include non-English and non-digital components, and integration of services that proactively reach underrepresented populations. Specifically, findings suggested translations of SDM measurement instruments, using interpreters and supported completion of the electronic system (e.g., on tablet computers in clinic, supported by staff/family) to meet inclusivity and accessibility needs and maximise engagement."*

**COMMENT 1.5**

5. In relation to the point above: the authors state that they did not record participant ethnicity, yet report that all patient participants were " from a white British background". Please clarify (p12, line 45).

**RESPONSE 1.5**

Thank you for highlighting this discrepancy. This is now corrected and ethnicity included in the list of socio-demographic characteristics considered important a priori. We have clarified this

on p.8 and the section now reads: *"Sampling characteristics considered a priori included variation by [...] iii) socio-demographic characteristics (i.e. sex, age, ethnicity).*

There were, however, challenges in obtaining consistent ethnicity data from hospital records which meant that data needed to be collected retrospectively, during the interview. This meant that the use of ethnicity as a sampling criterion was not possible. We have added more detail to this limitation in the discussion and the relevant section now reads: *"Recruitment of patient participants from a more diverse of population groups was hampered because hospital electronic patient record systems did not comprehensively recording ethnicity data. This meant that ethnicity data was not used as an a priori sampling criterion but collected retrospectively."*

**COMMENT 1.6**

6. Table 2: The summaries describing the themes "Complexities of SDM measurement" and "Challenges of distributed decision making" seem to overlap, both refer to how hard it may be to interpret SDM results. The same holds for the summaries describing the themes 'Approaches to responding to feedback' and 'Approaches to providing feedback to clinical teams'. Please make distinction more clear, or collapse themes. Note that in both cases, the distinction between the themes is clear in the text (paragraphs 1.2-1.3, 2.1-2.2).

**RESPONSE 1.6**

We appreciate the reviewer's suggestions. We agree that the summaries in Table 2 did not sufficiently differentiate between these themes. In response, we have revised the summaries to better reflect the unique focus of each theme, which describe

- core issues around how to measure SDM effectively and reliably (theme 1.2) and identifying where and how SDM problems occurs in complex care pathways (theme 1.3),
- how feedback about patients SDM scores is received by clinical teams and by whom exactly (theme 2.2) and what happens after feedback is received and how patients are involved (theme 2.3)

| **Themes** | **Summary of findings** |
|---|---|
| 1.2 Complexities of SDM measurement | Both participant groups discussed the perceived complexities of reliably measuring SDM and pointed out common sources of variability of SDM experiences that might influence patient-reported SDM scores. |
| 1.3 Challenges of distributed decision making | Some participants in both participant groups discussed difficulties in tracking and attributing SDM experiences in complex care pathways involving multiple healthcare providers. |
| 2.2 Approaches to providing feedback to clinical teams | Professionals expressed their preference for a 'triage process' to providing feedback to clinical teams, whereby data about patient-reported SDM scores are initially processed and filtered by intermediaries before involving surgeons, only where necessary. |
| 2.3 Approaches to responding to feedback | Both participant groups considered a follow-up encounter to be an appropriate response when patient-reported SDM scores suggest deficiencies in SDM experiences, emphasising that tailored discussions need to address individual concerns. |

**COMMENT 1.7**

7. P21, lines 48-52: The quote by PAT017 is telling about how this patient (and maybe others too) view as the core task of a surgeon: “I think it's a bit much to expect to speak to the surgeon, I know that's what they're there for, but you don't want to take them away from what they’re meant to be doing. [...].” (PAT017)
This is an essential point with regard to the whole endeavor of trying to improve SDM in this setting: to what extent is there buy-in both from patients and healthcare professionals, that SDM is worth improving since it may be what they (patient together with the surgeon) are meant to be doing. If not, what then is the point of providing this feedback to surgeons? I invite the authors to consider to what extent similar ideas about roles were expressed by their participants and if the implications may be worth discussing.

**RESPONSE 1.7**

The reviewer makes a good point and we agree that this particular finding warrants more attention. We have expanded on this result in our discussion section and have included implications for practice. The section now reads:

*“Findings suggest that the implementation of real-time SDM monitoring and feedback is a service-wide responsibility. Results highlighted the need for careful consideration of core responsibilities and complexities when remediating patient-reported deficiencies in SDM. Both professional and patient participants noted that surgeons are not always the most appropriate to lead SDM improvements, and discussed the potential suitability of other members of the clinical or departmental team members (e.g. nurses, administrative staff) in certain contexts. Careful consideration should therefore be given to identify appropriate professional roles for receiving feedback and defining new processes for how services should respond. [66,67][68–70] Results highlight the need to co-design approaches to ensure tailored responses to SDM feedback are acceptable to patients and professionals. This includes identifying suitable roles within each surgical department, defining new responsibilities collaboratively with teams, and co-developing supportive materials (e.g. new standard operating procedures) to ensure seamless integration into existing workflows. Substantial challenges of widely adopting SDM in clinical practice, however, have been noted [3,14,15] and various facilitators and barriers to implementation have been identified [16–18]. Any programmes that aim to routinely embed SDM monitoring and feedback should build on existing knowledge about mechanisms that support the adoption of SDM in practice [19–21] and may utilise strategies for embedding SDM into care pathways at varying levels, including micro (individual), meso (departmental), and macro (organisational) levels [22]. Established frameworks from behavioural and implementation sciences (e.g., Theoretical Domains Framework, Normalisation Process Theory [23,24]) could help in developing approaches systematically and transparently which incorporate relevant stakeholder insights and map context-specific barriers and facilitators. Additionally, more work is needed to develop effective strategies for stakeholder engagement that can address identified healthcare professional concerns (e.g., worries about time pressures, performance evaluation or scrutiny) to maximise uptake of the future intervention to improve SDM.”*

**COMMENT 1.8**

8. P26, line 28-32: The authors state that "This study provides an important first step to addressing the UK National Institute for Health and are Excellence call for research into new interventions targeting inclusive, sustained, health system-level SDM improvement [10]." However, as they have noted before, their real-time SDM monitoring was perceived to be limited in its inclusivity, an issue of particular importance to patient. Also, they note a lack of diversity in their sample, in terms e.g. of ethnicity (although this was stated not to have been measured), including only patients who had completed SDM monitoring, using convenience sample of healthcare professionals, and distributing only an English version of the SDM-survey. Please provide an explicit rationale for why the present system nonetheless may be inclusive.

**RESPONSE 1.8**

We thank the reviewer for pointing out this important point and we acknowledge important limitations regarding inclusivity in the present study and have made significant changes in the manuscript. We have moved the paragraph where we describe future research to explore inclusivity to the "Implications for research" section and have expanded on how the present system addresses some inclusivity needs. The relevant section reads:

*"The use of digital systems to monitor patient outcomes in surgery has accelerated since the COVID-19 pandemic, but the need to prioritise health equity and research to improve language and digital inclusivity is widely acknowledged [25]. A related study has found a range of factors affecting use and engagement with the system for real-time SDM monitoring [26]. Patients particularly valued its low burden and ease of use, noting that the clear, accessible design supported participation across a diverse range of users, including those with varying levels of health literacy and digital confidence."*

Whilst this study sample lacks diversity, our wider programme includes a dedicated line of work that aims to capture views from population groups which experience intersecting inequalities, and who therefore are also likely to be disproportionately affected by poor SDM. In response to comment 1.4, we have included detailed explanations of how complementary work aims to address the current limitations and how it may optimise inclusivity, including, for example, translations of the SDM survey into multiple languages, and offering non-digital versions.

**COMMENT 1.9**

Textual:
P 7, line 8-9. It seems a bit odd to use the Cochrane review by Légaré et al (2018) as evidence to support that SDM can impact health outcomes. Please review and adapt.

**RESPONSE 1.9**

Thank you for this observation. We included the Cochrane systematic review as evidence because it synthesised studies that evaluated a range of interventions for increasing SDM and summarised the effect sizes for a variety of primary and secondary outcomes chosen by the large body of literature. The particular health outcomes identified in the review included Health-related quality of life, health status, anxiety, depression or stress. We therefore felt it was appropriate to reference this systematic review. To improve the supporting evidence for this statement, we have now added example references which presented the primary results for

improved health outcomes linked to SDM interventions and have included further references (listed below) that explicitly investigated the link between SDM and health outcomes.

- Hughes TM, Merath K, Chen Q, Sun S, Palmer E, Idrees JJ, Okunrintemi V, Squires M, Beal EW, Pawlik TM. Association of shared decision-making on patient-reported health outcomes and healthcare utilization. Am J Surg. 2018 Jul;216(1):7-12. doi: 10.1016/j.amjsurg.2018.01.011. Epub 2018 Jan 31. PMID: 29395026.
- Shay LA, Lafata JE. Where is the evidence? A systematic review of shared decision making and patient outcomes. *Medical Decision Making* 2015 Jan;35(1):114-31. [DOI: 10.1177/0272989X14551638]
- Bieber C, Nicolai J, Gschwendtner K, Müller N, Reuter K, Buchholz A, Kallinowski B, Härter M, Eich W. How does a shared decision-making (SDM) intervention for oncologists affect participation style and preference matching in patients with breast and colon cancer?. Journal of Cancer Education. 2018 Jun;33:708-15.

**COMMENT 1.10**

P9, line 38-39: The third eclusion criterion is unclear, please rephrase.
P9, line 40-41: It is unclear what "those" refer to in "A sub-set of those patients were sampled…], since immediately before the authors have described which patients have been excluded.

**RESPONSE 1.10**

We thank the reviewer for the helpfully pointing out the inconsistencies and we agree that the wording was confusing. We have added detail to explain the exclusion criterion and have removed irrelevant and confusing wording related to sampling of participants from this eligibility section.

The exclusion criteria section now reads: *"Patients were excluded if they lacked decisional capacity for medical treatments, had undergone unplanned (emergency) surgery or were booked for outpatient procedures (e.g. gastrointestinal and urological endoscopy) because these decision contexts differ from those typically required to achieve high-quality SDM, including the ability and time for deliberation, the availability of multiple options and the involvement of both the patient and professional in making the treatment choice [1]."*

**RESPONSE 1.11**

P9, line 50-52: What do the authors mean by "Participant characteristics were assessed as the study progressed and recruitment efforts targeted under-represented patients."? Please clarify.

**RESPONSE 1.12**

Thank you. We have now amended this sentence to clarify that we intended to review sample characteristics as the study progressed to adjust recruitment efforts to purposively sample participants that are not already represented in the study.

This sections now reads: *"Available socio-demographic characteristics of participants were reviewed as recruitment progressed to adjust recruitment efforts to purposively sample individuals with different experiences, offering potentially varied perspectives."*

**COMMENT 1.11**

P10, line 12-16: The sentence "This involved multi-disciplinary ... more details) [31–33]." Does not read fully well, please correct.

**RESPONSE 1.13**

Thank you very much and we agree that changes to the wording were required to improve comprehensibility. The sentence now reads: "*This decision was supported by regular multi-disciplinary team discussions which involved reviewing whether the identified codes/themes provided sufficient conceptual depth to answer the research question*"

**COMMENT 1.123**

P21, line 20-21: What is the distinction between "conversation" and "consultation encounter"? Please explain.

**RESPONSE 1.143**

Thank you for querying this and we acknowledge that the terms used are synonymous and have consolidated this to improve clarity.

The sentence now reads: *"All participants indicated that a process which provided a follow-up encounter (e.g., another consultation discussion) would be acceptable to them to address deficiencies in patient-reported SDM experiences."*

**Reviewer: 2**
Dr. Justin Clapp, University of Pennsylvania
Comments to the Author:
Thanks for the opportunity to review this manuscript. The authors report a qualitative interview study of patients and surgeons about a system for real-time patient reporting on shared decision making (SDM). The manuscript is clearly written, the methods are comprehensively laid out, and the results are well organized and supported nicely with many illustrative quotes. The sample characteristics aren't perfect but the authors appropriately acknowledge this in the limitations.

We thank the reviewer for their helpful comments to improve our manuscript which we have addressed in our revised version. We appreciate the reviewer considering our manuscript "clearly written" and our methods "comprehensively laid out" and expressing support for our research project.

**COMMENT 2.1**

I have one major piece of feedback. The results are quite long for a clinical journal—to the point where I'd strongly consider slimming them down to the themes the authors find most important/impactful.

**RESPONSE 2.1**

We acknowledge the reviewers comment regarding the length of the results and have reviewed sections carefully and reduced and consolidated content throughout the results section where possible.

We agree that substantially shorter manuscripts are generally preferred by clinical audiences. Considering the wider BMJ Open audience and qualitative research standards, however, we hope to have satisfied the needs for a variety of readers. The inclusion of Table 2 aimed to provide a concise summary of findings for each theme, intended to convey the key findings without the need to read in-depth descriptions and exemplary quotes. We prefer to keep a certain level of length and depth, because some readers might be interested in the detailed findings and would like to engage with the longer, supporting quotations to put the discussion points into context. The level of detail in the manuscript aligns with similar multi-stakeholder explorative work published in BMJ Open [27,28] and we are also happy to take editorial guidance on this matter.

**COMMENT 2.2**

Meanwhile, I didn't find the discussion to offer a terribly robust engagement with the findings. I'd suggest extending the discussion and shortening the results. The interviews have revealed some issues that have quite interesting implications for the attempt to characterize SDM and report it to practitioners. For example, the surgeons questioned whether patient-reported experiences actually reflect the quality of surgeons' communication. They have a good point here: tools for the measurement of SDM (observational and patient-reported) have not been found to have good correspondence in several studies. In my experience doing ethnographic work combining interviewing and observation, I've similarly found that the degree to which patients describe being involved in a consultation often is not apparent in the consultation itself. The points that patient and surgeon interviewees made about the distributed nature of decision making also have major implications for any effort to characterize and report SDM. Since in many areas of medicine care is increasingly done via multidisciplinary teams, entailing chains of clinical interactions, it's possible that various elements of SDM are being accomplished at various stages. I'd like to see the authors address these kinds of issues at greater length in the discussion, engaging with relevant literature; I think it'd substantially enrich the manuscript.

**RESPONSE 2.2**

Thank you for this important feedback and helpful suggestions to enhance our discussion. We have significantly extended our discussion to address these, also taking into account Reviewer 1 comments.

Specifically, we have added content to the 'Implications for research' section to discuss results related to distributed decision-making and measurement incongruence. The sections now read:

*"SDM is widely recognised as a multi-faceted process with various conceptual models describing the steps to be taken, or core elements necessary to achieve high-quality SDM [29]. Importantly, SDM can be an ongoing process and "distributed over time, place, and between*

*different participants" [30]. Participants narratives in this study reflected this complexity, describing multiple interactions with different healthcare providers, including primary care and peri-operative services. Interactions during any of these encounters are likely to contribute to the collective process of decision-making and affect patients' overall SDM experience [31]. Distributed decision-making has also shown to exclude patients from involvement in decision-making [32]. These dynamics present challenges for capturing patient-reported SDM experience at a single, fixed timepoint and underscore existing research gaps regarding the optimal timing of SDM measurement [5,33]. Routine SDM measurement following a surgical consultation may only represent a narrow snapshot, or would give little insight into deficiencies that arose earlier in the SDM process [34]. Addressing this limitation may require a deeper understanding of individual patient pathways and personal contexts, as well as more collaborative, longitudinal approaches to SDM assessment to identify how patients develop informed preferences. There is, however, a lack of evidence to understand how SDM can be effectively supported across varying time, places and participants. Some SDM models have taken distributed decision-making into account during their theoretical development [35] and emphasise the need for practical guidance for clinicians to incorporate, for example, diverse information sources and patients' social networks into the decision-making processes [36,37]. Some more radical suggestions include restructuring consultations to include perspectives from a range of healthcare providers [32]. There are important future research opportunities to explore how SDM measurement can be optimised and how SDM can be supported in the context of distributed decision-making in surgery."*

And

*"Concerns regarding the reliability and validity of SDM data were prominent in this study, affecting acceptability of routine SDM monitoring and feedback in particular among professionals. These concerns reflect ongoing debates in the field where significant challenges in SDM measurement quality have been well documented [5,33,38]. Many existing instruments lack high quality psychometric and methodological foundations [39] and studies have highlighted misalignment between patient, provider and observer assessment of SDM quality [40,41]. Such discrepancies raise important questions about conceptual deficits and valid perspectives in SDM measurement that warrant attention in future research."*

**References**


1 van der Horst DEM, Garvelink MM, Bos WJW, *et al.* For which decisions is Shared Decision Making considered appropriate? – A systematic review. *Patient Educ Couns*. 2023;106:3–16. doi: 10.1016/J.PEC.2022.09.015

2 Hoffmann C, Avery KNL, Macefield RC, *et al.* Real-time monitoring and feedback to improve shared decision-making for surgery (the ALPACA Study): protocol for a mixed-methods study to inform co-development of an inclusive intervention. *BMJ Open*. 2024;14. doi: 10.1136/BMJOPEN-2023-079155

3 National Institute for Health and Care Excellence. Shared decision making. NICE guideline [NG197]. 2021.

4 National Institute for Health and Care Excellence. Shared decision making [A] Evidence review for effectiveness of approaches and activities to increase engagement in shared decision making and the barriers and facilitators to engagement. Published Online First: 2021.

5 National Institute for Health and Care Excellence. Shared decision making [B] Evidence review for interventions to support effective shared decision making. 2021.

6 Ivers N, Jamtvedt G, Flottorp S, *et al.* Audit and feedback: Effects on professional practice and healthcare outcomes. *Cochrane Database of Systematic Reviews*. 2012;2012. doi: 10.1002/14651858.CD000259.pub3

7 Lee R, Baeza JI, Fulop NJ. The use of patient feedback by hospital boards of directors: a qualitative study of two NHS hospitals in England. *BMJ Qual Saf*. 2018;27:103–9. doi: 10.1136/BMJQS-2016-006312

8 NIHR Dissemination Centre. Improving Care by Using Patient Feedback. *Health and Social Care Services Research*. Published Online First: December 2019. doi: 10.3310/THEMEDREVIEW-04237

9 Absolom K, Warrington L, Hudson E, *et al.* Phase III Randomized Controlled Trial of eRAPID: eHealth Intervention During Chemotherapy. *J Clin Oncol*. 2021;39:734–47. doi: 10.1200/JCO.20.02015

10 Sheard L, Peacock R, Marsh C, *et al.* What's the problem with patient experience feedback? A macro and micro understanding, based on findings from a three-site UK qualitative study. *Health Expectations*. 2019;22:46–53. doi: 10.1111/HEX.12829

11 Flott KM, Graham C, Darzi A, *et al.* Can we use patient-reported feedback to drive change? the challenges of using patient-reported feedback and how they might be addressed. *BMJ Qual Saf*. 2017;26:502–7. doi: 10.1136/BMJQS-2016-005223

12 Brown B, Gude WT, Blakeman T, *et al.* Clinical Performance Feedback Intervention Theory (CP-FIT): A new theory for designing, implementing, and evaluating feedback in health care based on a systematic review and meta-synthesis of qualitative research. *Implementation Science*. 2019;14:1–25. doi: 10.1186/S13012-019-0883-5/TABLES/10

13 Islam S, Joseph O, Chaudry A, *et al.* "We are not hard to reach, but we may find it hard to trust" .... Involving and engaging 'seldom listened to' community voices in clinical translational health research: a social innovation approach. *Research Involvement and Engagement 2021 7:1*. 2021;7:1–15. doi: 10.1186/S40900-021-00292-Z

14 Bravo P, Härter M, McCaffery K, *et al.* Editorial: 20 years after the start of international Shared Decision-Making activities: Is it time to celebrate? Probably.... *Z Evid Fortbild Qual Gesundhwes*. 2022;171:1–4. doi: 10.1016/J.ZEFQ.2022.05.009

15 Frosch DL, Moulton BW, Wexler RM, *et al.* Shared decision making in the United States: policy and implementation activity on multiple fronts. *Z Evid Fortbild Qual Gesundhwes*. 2011;105:305–12. doi: 10.1016/J.ZEFQ.2011.04.004

16 Légaré F, Ratté S, Gravel K, *et al.* Barriers and facilitators to implementing shared decision-making in clinical practice: Update of a systematic review of health professionals' perceptions. *Patient Educ Couns*. 2008;73:526–35. doi: 10.1016/J.PEC.2008.07.018

17 Alsulamy N, Lee A, Thokala P, *et al.* What Influences the Implementation of Shared Decision Making: An Umbrella Review. *Patient Educ Couns*. 2020;103:2400–7. doi: 10.1016/J.PEC.2020.08.009

18 Légaré F, Witteman HO. Shared Decision Making: Examining Key Elements And Barriers To Adoption Into Routine Clinical Practice. *https://doi.org/101377/hlthaff20121078*. 2017;32:276–84. doi: 10.1377/HLTHAFF.2012.1078

19 Joseph-Williams N, Lloyd A, Edwards A, *et al.* Implementing shared decision making in the NHS: lessons from the MAGIC programme. *BMJ*. 2017;357:j1744. doi: 10.1136/bmj.j1744

20 Davies C, Fattori F, O'Donnell D, *et al.* What are the mechanisms that support healthcare professionals to adopt assisted decision-making practice? A rapid realist review. *BMC Health Serv Res*. 2019;19:1–14. doi: 10.1186/S12913-019-4802-X/TABLES/4

21 Waldron T, Carr T, McMullen L, *et al.* Development of a program theory for shared decision-making: A realist synthesis. *BMC Health Serv Res*. 2020;20:1–17. doi: 10.1186/S12913-019-4649-1/TABLES/4

22 Holm A, Rodkjær LØ, Bekker HL. Integrating Patient Involvement Interventions within Clinical Practice: A Mixed-Methods Study of Health Care Professional Reasoning. *MDM Policy Pract*. 2024;9. doi: 10.1177/23814683241229987/ASSET/IMAGES/LARGE/10.1177_23814683241229987-FIG4.JPEG

23 Murray E, Treweek S, Pope C, *et al.* Normalisation process theory: A framework for developing, evaluating and implementing complex interventions. *BMC Med*. 2010;8:1–11. doi: 10.1186/1741-7015-8-63/TABLES/3

24 Francis JJ, O'Connor D, Curran J. Theories of behaviour change synthesised into a set of theoretical groupings: introducing a thematic series on the theoretical domains framework. *Implementation Science*. 2012;7:1–9. doi: 10.1186/1748-5908-7-35/TABLES/2

25 Fong Y, Melstrom L, Evans HL. Practical Guide to Remote Patient Monitoring in Surgical Patients. *JAMA Surg*. Published Online First: 8 January 2025. doi: 10.1001/JAMASURG.2024.6015

26 Hoffmann C, Avery K, Macefield R, *et al.* Usability of an Automated System for Real-Time Monitoring of Shared Decision-Making for Surgery: Mixed Methods Evaluation. *JMIR Hum Factors*. 2024;11:e46698. doi: 10.2196/46698

27 Hanley J, Ure J, Pagliari C, *et al.* Experiences of patients and professionals participating in the HITS home blood pressure telemonitoring trial: a qualitative study. *BMJ Open*. 2013;3:e002671. doi: 10.1136/BMJOPEN-2013-002671

28 Cullen L, Grenfell P, Rodger A, *et al.* 'Just another vial...': a qualitative study to explore the acceptability and feasibility of routine blood-borne virus testing in an emergency department setting in the UK. *BMJ Open*. 2019;9:e024085. doi: 10.1136/BMJOPEN-2018-024085

29 Bomhof-Roordink H, Gärtner FR, Stiggelbout AM, *et al.* Key components of shared decision making models: A systematic review. BMJ Open. 2019;9.

30 Elwyn G. Shared decision making: What is the work? *Patient Educ Couns*. 2021;104:1591–5. doi: 10.1016/J.PEC.2020.11.032

31 Rapley T. Distributed decision making: The anatomy of decisions-in-action. *Sociol Health Illn*. 2008;30:429–44. doi: 10.1111/J.1467-9566.2007.01064.X,

32 Johnson R, Turner K, Feder G, *et al*. Shared decision making in consultations for hypertension: Qualitative study in general practice. *Health Expectations*. 2021;24:917–29. doi: 10.1111/HEX.13234;REQUESTEDJOURNAL:JOURNAL:13697625;JOURNAL:JOURNAL:13697625;WGROUP:STRING:PUBLICATION

33 Sepucha KR, Breslin M, Graffeo C, *et al*. State of the Science: Tools and Measurement for Shared Decision Making. *Academic Emergency Medicine*. 2016;23:1325–31. doi: 10.1111/ACEM.13071;JOURNAL:JOURNAL:15532712;WGROUP:STRING:PUBLICATION

34 Ofstad EH, Frich JC, Schei E, *et al*. Temporal characteristics of decisions in hospital encounters: A threshold for shared decision making? A qualitative study. *Patient Educ Couns*. 2014;97:216–22. doi: 10.1016/J.PEC.2014.08.005

35 Elwyn G, Lloyd A, May C, *et al*. Collaborative deliberation: A model for patient care. *Patient Educ Couns*. 2014;97:158–64. doi: 10.1016/J.PEC.2014.07.027

36 Entwistle VA, France EF, Wyke S, *et al*. How information about other people's personal experiences can help with healthcare decision-making: A qualitative study. *Patient Educ Couns*. 2011;85. doi: 10.1016/J.PEC.2011.05.014

37 Bussey LG, Sillence E. The role of internet resources in health decision-making: a qualitative study. *Digit Health*. 2019;5:2055207619888073. doi: 10.1177/2055207619888073

38 Barr PH Psych PJ, Elwyn MB BCh GB, Professor A, *et al*. Measurement challenges in shared decision making: putting the 'patient' in patient-reported measures. *Health Expectations*. 2016;19:993–1001. doi: 10.1111/HEX.12380

39 Gärtner FR, Bomhof-Roordink H, Smith IP, *et al*. The quality of instruments to assess the process of shared decision making: A systematic review. *PLoS One*. 2018;13. doi: 10.1371/journal.pone.0191747

40 Entwistle VA, Watt IS, Gilhooly K, *et al*. Assessing patients' participation and quality of decision-making: insights from a study of routine practice in diverse settings. *Patient Educ Couns*. 2004;55:105–13. doi: 10.1016/J.PEC.2003.08.005

41 Kasper J, Heesen C, Köpke S, *et al*. Patients' and Observers' Perceptions of Involvement Differ. Validation Study on Inter-Relating Measures for Shared Decision Making. *PLoS One*. 2011;6:e26255. doi: 10.1371/JOURNAL.PONE.0026255

---

## VERSION 2 - REVIEW

---

| | |
|---|---|
| **Reviewer** | **1** |
| **Name** | **Pieterse, Arwen** |
| **Affiliation** | **Leiden University Medical Center, Biomedical Data Sciences** |
| **Date** | **03-Jun-2025** |

## COI

---

Thank you for the opportunity to review a revision of the manuscript. The authors have thoughtfully and comprehensively addressed the reviewers' comments. I have no further comments, except for a sentence that needs rewriting (se below) and I wonder if the page numbers included in the COREQ checklist are accurate, please review. E.g., Sample size and Description of the sample can be found on p. 11 (bottom of page) or 13 (top left of page) depending on the page number used, not on p. 10.

Textual:

p. 27, lines 49-54: The following sentence (providing helpful clarification in response to a comment on the previous version) needs rewording as it does not read fully well:
"Recruitment of patient participants from a more diverse of population groups was hampered because hospital electronic patient record systems did not comprehensively recording ethnicity data."
